# MISP Suppresses Ferroptosis via MST1/2 Kinases to Facilitate YAP Activation in Non‐Small Cell Lung Cancer

**DOI:** 10.1002/advs.202415814

**Published:** 2025-02-28

**Authors:** Fuquan Zhang, Bingtao Huang, Yiming Xu, Guohong Cao, Mingjing Shen, Changmin Liu, Judong Luo

**Affiliations:** ^1^ Department of Thoracic and Cardiovascular Surgery The Second Affiliated Hospital of Nantong University The First People's Hospital of Nantong Nantong 226000 China; ^2^ Department of Thoracic Surgery Binzhou Medical University Hospital Binzhou 256600 China; ^3^ Department of Cardiothoracic Surgery The Second Affiliated Hospital of Soochow University Suzhou 215004 China; ^4^ Department of Radiation Oncology The First Affiliated Hospital of USTC Division of Life Sciences and Medicine University of Science and Technology of China Hefei Anhui 230001 China; ^5^ Department of Radiation Oncology Anhui Provincial Cancer Hospital Hefei Anhui 230031 China; ^6^ Department of Radiotherapy Tongji Hospital School of Medicine Tongji University Shanghai 200065 China

**Keywords:** ferroptosis, MISP, MST kinases, SLC7A11, YAP

## Abstract

Despite advances in non‐small cell lung cancer (NSCLC) therapies, resistance remains a major challenge. Ferroptosis, a form of regulated cell death, plays a key role in cancer progression and treatment response. However, the mechanisms governing ferroptosis in NSCLC are not fully understood. The Hippo pathway, which regulates cell proliferation, has recently been implicated in ferroptosis regulation. In this study, we identify Mitotic Spindle Positioning (MISP) as a critical inhibitor of ferroptosis in NSCLC. MISP is upregulated in NSCLC tissues, and its loss sensitizes cells to ferroptosis, reducing cell proliferation in vitro and in vivo. Mechanistically, MISP binds to the SARAH domain of MST1/2 kinases, inhibiting their homodimerization and autophosphorylation, leading to sustained activation of YAP, a transcriptional coactivator in the Hippo pathway. YAP activation increases SLC7A11 expression, which protects cells from ferroptosis. We also identify a mutant MISP‐R390/391A that disrupts MISP‐MST1/2 binding, further illustrating the MST1/2‐dependent inhibition of Hippo signaling. Notably, MISP is a target of YAP, creating a feedback loop that amplifies YAP signaling. Our findings suggest a novel MISP‐YAP axis regulating ferroptosis, positioning MISP as a potential therapeutic target for NSCLC, especially in cases with dysregulated YAP.

## Graphical abstract

This study explores the role of Mitotic Spindle Positioning (MISP) as a key inhibitor of ferroptosis in non‐small cell lung cancer (NSCLC). MISP upregulation in NSCLC tissues leads to ferroptosis resistance, promoting cell proliferation. Mechanistically, MISP suppresses Hippo signaling via MST1/2 kinases, activating YAP and enhancing SLC7A11 expression, which protects cells from ferroptosis. The discovery of a mutant MISP variant further elucidates the MISP‐YAP axis and its therapeutic implications in NSCLC, particularly for YAP‐dysregulated cases.

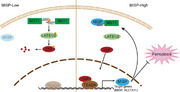

## 1 Introduction

The Hippo pathway controls a diverse array of physiological processes, such as organ size and tissue homeostasis.^[^ ^1^ ^]^ Central to this pathway is a kinase cascade, wherein the mammalian STE20‐like protein kinases (MST1/2) phosphorylate the large tumor suppressor kinases (LATS1/2) in a complex with the scaffold proteins Salvador homolog 1 (SAV1) and MOB kinase activator 1A/B (MOB1A/B). The LATS1/2 kinases then phosphorylate and restrain Yes‐associated protein (YAP1), and its paralog transcriptional coactivator with PDZ‐binding motif (TAZ), the major downstream effectors that bind TEADs to stimulate numerous target genes, such as *CTGF*, *CYR61*, *BIRC5*, and *ANKRD1*.^[^ ^2^ ^]^ The Hippo signaling has emerged as a critical cancer signaling network and is altered in human cancers.^[^ ^1^ ^]^ Although it is considered to be a promising therapeutic target for cancers, perturbation of the Hippo pathway remains challenging, owing to limited targets, such as YAP and TEADs.^[^ ^3^ ^]^ MST1/2 is essential for YAP/TAZ activity, and loss of MST1/2 results in cell hyperproliferation and tumorigenesis.^[^ ^4^ ^]^ However, mutations in MST1/2 kinases have not been commonly found in human cancers, and it is not yet known how MST1/2 activity is diminished in cancers. Understanding how their tumor‐suppressor activities are regulated would be helpful in expanding the potential of targeting MST1/2 in the Hippo signaling pathway for cancer therapy.

Unlike other signaling pathways, which are activated by specific ligands and are crucial for tissue development, the Hippo pathway is unique in its ability to sense a variety of environmental cues.^[^ ^5^ ^,‐^ ^7^ ^]^ This includes actin cytoskeleton remodeling, a process mediated by the accumulation of filamentous actin (F‐actin).^[^ ^8^, ^9^, ^10^ ^]^ Mitotic Spindle Positioning (MISP), which contains multiple actin‐binding sites, has recently been identified as a novel actin‐related protein that plays a significant role in cellular cytoskeletal reorganization.^[^ ^11^, ^12^, ^13^ ^]^ However, its role in cancers, including lung cancer, remains poorly understood.

The Hippo signaling pathway is notorious for contributing to therapy resistance,^[^ ^14^, ^15^, ^16^ ^]^ and serves as a crucial regulator of ferroptosis,^[^ ^17^, ^18^, ^19^ ^]^ a unique type of cell death triggered by iron accumulation and lipid peroxidation, which has been extensively studied in cancers.^[^ ^20^, ^21^ ^]^ Solute carrier family 7‐member 11 (SLC7A11, also known as xCT) is the primary transporter responsible for importing extracellular cystine into the cytosol for glutathione (GSH) synthesis, playing an essential role in protecting against ferroptosis.^[^ ^22^ ^]^ Deregulation of SLC7A11 has been observed in various human cancers.^[^ ^23^, ^24^ ^]^ Understanding the mechanisms that regulate ferroptosis in cancers may provide potential strategies for activating ferroptosis and overcoming therapeutic resistance.

In our study, MISP was identified as a critical driver of ferroptosis resistance that facilitates lung cancer cell proliferation, consistent with its significant upregulation in lung cancer tissues. As a new component of the Hippo signaling pathway, MISP interacts with MST1/2, disrupting the assembly of MST homodimers, leading to YAP translocation and subsequent stimulation of SLC7A11 expression, a potent inhibitor of ferroptosis. Our findings uncover the MISP‐MST1/2‐SLC7A11 axis as a key mediator in lung cancer progression and provide support for the connection between MISP and ferroptosis, offering significant therapeutic implications for YAP‐driven tumors.

## 2 Results

### 2.1 MISP is Altered in Lung Cancer Tissues

**Figure 1:**
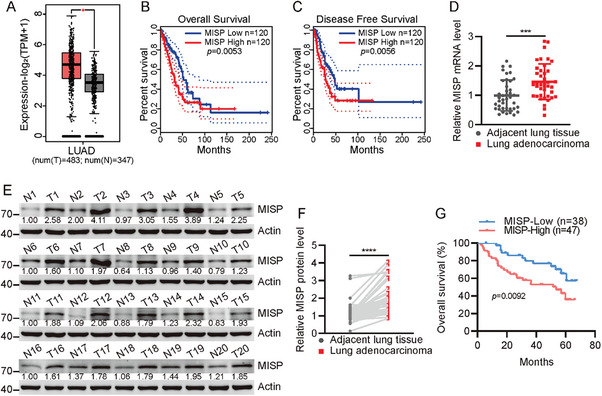
MISP is upregulated in NSCLC. A) Scatter plot showing MISP expression in NSCLC and adjacent tissues from the TCGA dataset. The figure was generated using GEPIA (http://gepia.cancer‐pku.cn/). *n* = 347 (adjacent) and *n* = 483 (NSCLC) in TCGA‐LUAD. B) Survival analysis of NSCLC patients from TCGA dataset stratified by top 25% quarter of low and high MISP expression. The figure was generated using GEPIA (http://gepia.cancer‐pku.cn/). C) Disease‐free survival analysis in NSCLC patients from the TCGA dataset stratified by top 25% quarter of low and high MISP expression. The figure was generated using GEPIA (http://gepia.cancer‐pku.cn/). D) Quantitative PCR analysis of MISP expression in NSCLC and adjacent tissues. *n* = 40 (adjacent) and *n* = 40 (NSCLC). E) Immunoblot analysis of MISP expression in NSCLC and adjacent tissues (cases 1 to 20). *n* = 20 (adjacent) and *n* = 20 (NSCLC). F) Protein quantification of MISP levels in E). Statistical significance was determined by a paired two‐tailed Student's *t*‐test as indicated. G) Kaplan‐Meier plots showing the overall survival of NSCLC patients with high or low MISP expression analyzed by qPCR. Data are presented as mean ± SEM. Unpaired *t*‐test was used in A and D to determine statistical significance. *p < 0.05, **p < 0.01, ***p < 0.001, ****p < 0.0001.

To assess the clinical relevance of MISP in lung cancers, MISP expression was analyzed in transcriptomic datasets from The Cancer Genome Atlas (TCGA), GSE32863, GSE33532, and GSE44077. We discovered that MISP was significantly upregulated in lung cancers relative to adjacent counterparts (**Figure** **1**A; Figure S1A, Supporting Information), and was correlated with shorter patient survival and disease‐free times (Figure 1B,C). An additional 40 paired lung cancer and their noncancerous tissues were used to confirm the observation. Both MISP mRNA and protein levels were dramatically increased in lung cancer tissues (Figure 1D–F; Figure S1B, Supporting Information), and elevated MISP predicted worse clinical outcomes in patients with lung cancer (Figure 1G). These results suggest a potential oncogenic role of MISP in lung cancer.

### 2.2 MISP Suppresses Ferroptosis to Promote Lung Cancer Cell Proliferation

**Figure 2:**
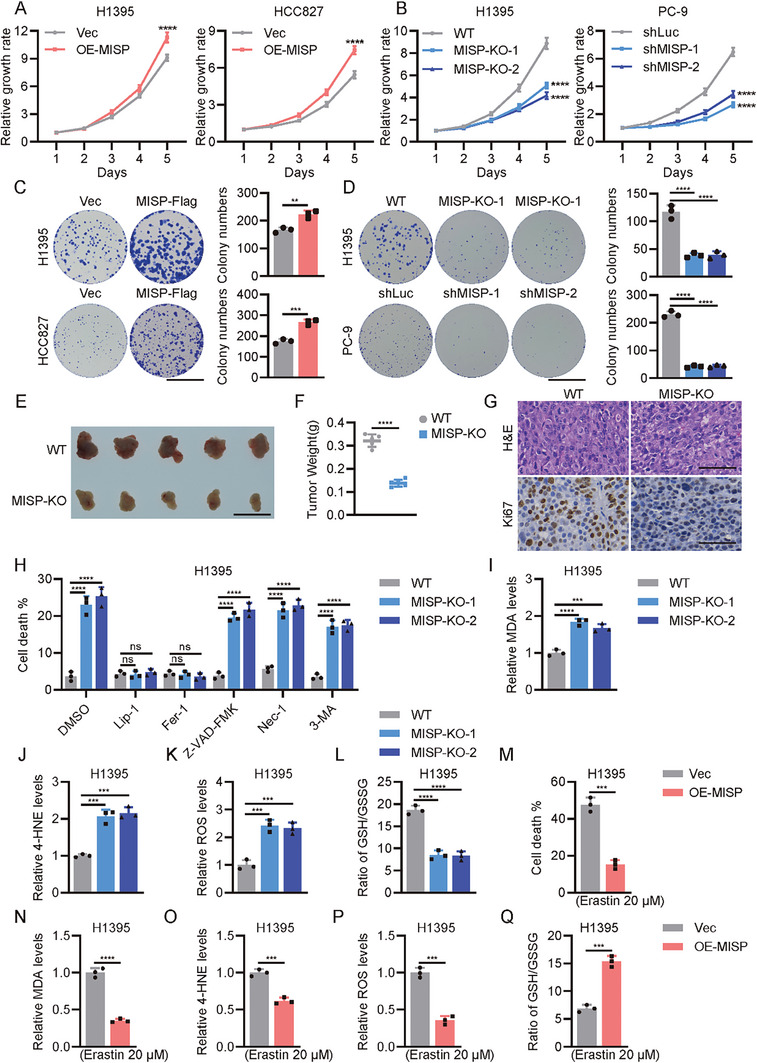
MISP drives resistance to ferroptosis in NSCLC cells. A) Determination of cell proliferation in H1395 and HCC827 cells upon MISP overexpression using the cell counting kit 8 (CCK8). B) Determination of cell proliferation in control and MISP‐depleted H1395 and PC‐9 cells by CCK8. C,D) Demonstration and quantification of colony formation abilities in indicated cells upon MISP overexpression C) and MISP ablation D). Scale bar, 10 mm. E) Gross image of xenografts from nude mice implanted with wild‐type (WT) and *MISP*‐deficient H1395 cells. Scale bar, 10 mm. F) Analysis of tumor weight from nude mice implanted with wild‐type (WT) and *MISP*‐deficient H1395 cells. G) Representative images of H&E and IHC staining of Ki67 in indicated tumor sections. Scale bar, 50 µm. H) Trypan blue staining and quantification of cell death in WT and MISP‐depleted cells treated with DMSO, Lip‐1 (500 nm), Fer‐1 (10 µM), Z‐VAD‐FMK (10 µM), Nec‐1 (10 µM) and 3‐MA (1 mM) for 24 h. I–K) Relative levels of MDA I), 4‐HNE J), and ROS K) in WT and MISP‐depleted H1395 cells. L) Determination of GSH and GSSG levels in indicated cells. M) Trypan blue staining and quantification of cell death in vector and MISP‐overexpressing cells treated with Erastin (20 µM) for 24 h. N–P) Relative levels of MDA N), 4‐HNE O), and ROS P) in vector and MISP‐overexpressing cells treated with Erastin (20 µM) for 24 h. Q) Determination of GSH and GSSG levels in vector and MISP‐overexpressing cells treated with Erastin (20 µM) for 24 h. Data are presented as mean ± SEM. One‐way ANOVA was used in A,B, D, and H–L to determine statistical significance. Unpaired *t*‐test was used in C, F, M–Q to determine statistical significance. *p < 0.05, **p < 0.01, ***p < 0.001, ****p < 0.0001.

To examine how elevated MISP expression in lung cancer affects its progression, several lung cancer cell lines were collected to test MSIP expression. PC‐9, H1395, and HCC827 cell lines were selected for functional investigation in vitro (Figure S2A,B, Supporting Information). MISP overexpression in H1395 and HCC827 cells resulted in marked cell proliferation (**Figure** **2**A; Figure S2C, Supporting Information), while either MISP knockout in H1395 or MISP depletion in PC‐9 impaired their proliferative capabilities (Figure 2B; Figure S2D, Supporting Information). Similar observations were seen in terms of colony formation abilities (Figure 2C,D). In agreement with its tumor‐promoting role in vitro, MISP loss in H1395 cells greatly repressed tumor growth in nude mice (Figure 2E), as evidenced by smaller tumor weight and diminished Ki‐67 reactivity in *MISP*‐deficient xenografts (Figure 2F,G). Given that neoplastic progression is the result of deregulated cell proliferation together with survival, and MISP did not significantly trigger cell cycle progression (Figure S2E, Supporting Information), we therefore determined whether MISP induces cell death. Indeed, MISP deficiency in H1395 or PC‐9 cells induced robust cell death compared with that in wild‐type cells (Figure S2F,G, Supporting Information). This effect was almost completely reversed by ferroptosis inhibitors Liproxstatin‐1 and Ferrostatin‐1, but not by other inhibitors of apoptosis (Z‐VAD‐FMK), necrosis (Necrostatin‐1), or autophagy (3‐MA) (Figure 2H; Figure S2H, Supporting Information). Consistently, MISP‐depleted H1395 cells showed increased malondialdehyde (MDA), 4‐hydroxynonenal (4‐HNE), ROS production, and depletion of intracellular GSH (Figure 2I–L), indicating lipid peroxidation, iron accumulation, and ferroptosis. A similar effect was also observed in additional PC‐9 cells with MISP ablation (Figure S2I–L, Supporting Information). Conversely, forced MISP expression in H1395 and HCC827 cells showed resistance to cell death induced by Erastin (Figure 2M; Figure S2M, Supporting Information), a canonical ferroptosis inducer, and reduced lipid peroxidation, iron accumulation, and ferroptosis (Figure 2N–Q; Figure S2N–Q, Supporting Information). Collectively, these data suggest that MSIP sustains lung cancer survival via ferroptosis inhibition.

### 2.3 MISP Stimulates SLC7A11 Expression via Hippo‐YAP Signaling

To elucidate the mechanisms through which MISP inhibits ferroptosis, we conducted an analysis of lung cancer tissue transcript datasets from The Cancer Genome Atlas (TCGA) to identify potential signaling pathways and key genes that connect MISP to ferroptosis. We found that the Hippo signaling pathway, which is crucial for cell proliferation and tissue homeostasis, showed a positive correlation with higher MISP expression in lung cancer patients, as evidenced by Gene Set Enrichment Analysis (GSEA) (Figure S3A, Supporting Information). This pathway has also been recognized as a significant regulator of ferroptotic cell death in cancers.^[^ ^17^, ^19^ ^]^ Importantly, ferroptosis, particularly SLC7A11, a known inhibitor of ferroptosis induced by oxidative stress, was found to be upregulated in patients with high MISP expression (Figure S3A, Supporting Information). This suggests a potential MISP‐SLC7A11 axis in the regulation of ferroptosis. We hypothesized that MISP might regulate SLC7A11‐mediated ferroptosis via the Hippo signaling pathway. Our experiments confirmed this hypothesis, as SLC7A11, along with CYR61 and CTGF—two established YAP target genes—were markedly downregulated in PC‐9 cells where MISP was deleted (Figure S3B,C, Supporting Information). This effect was not observed in cells lacking YAP (Figure S3D–H, Supporting Information), indicating that Hippo signaling is essential for the induction of SLC7A11 expression by MISP.

**Figure 3:**
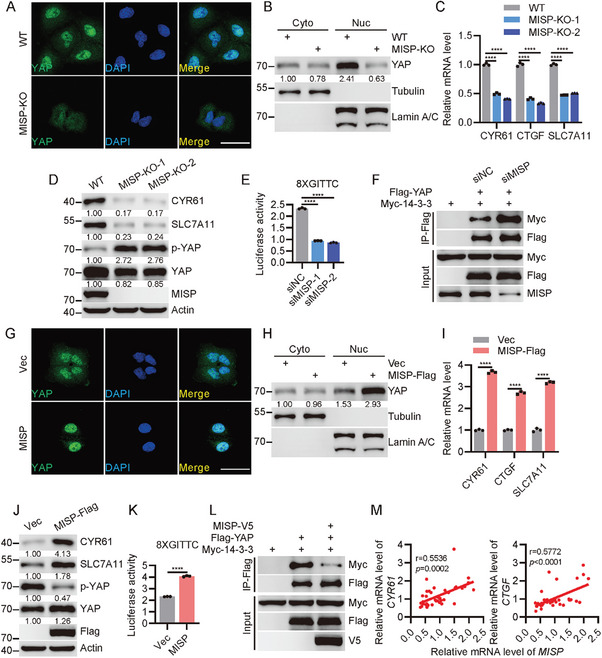
MISP stimulates SLC7A11 expression via Hippo signaling. A) Immunofluorescent staining showing the subcellular localization of YAP in WT and *MISP*‐deficient H1395 cells. Nuclei were stained with DAPI. Scale bar, 20 µm. B) Cytoplasmic and nuclear fractions derived from WT and *MISP*‐deficient H1395 cells were subjected to immunoblotting as indicated. α‐Tubulin and Lamin A/C were used as cytoplasmic and nuclear markers, respectively. C) qPCR evaluation of the transcriptional levels of SLC7A11 and YAP target genes in WT and *MISP*‐deficient H1395 cells. D) Immunoblot analysis of SLC7A11, YAP, YAP phosphorylation, and its target expression in WT and *MISP*‐deficient H1395 cells. E) Examination of TEAD luciferase reporter activity in 293T cells upon MISP knockdown. F) Association of YAP with 14‐3‐3 by immunoblot analysis. 293T cells were co‐transfected with Myc‐14‐3‐3 and vector or Flag‐YAP in the presence of control or small interfering RNA targeting MISP (siMISP). Co‐immunoprecipitates were detected using the indicated antibodies. G) Immunofluorescent staining showing the subcellular localization of YAP in vector and MISP‐overexpressing H1395 cells. Nuclei were stained with DAPI. Scale bar, 20 µm. H) Cytoplasmic and nuclear fractions derived from vector and MISP‐overexpressing H1395 cells were subjected to immunoblotting as indicated. I) qPCR evaluation of the transcriptional levels of SLC7A11 and YAP target genes in vector and MISP‐overexpressing H1395 cells. J) Immunoblot analysis of SLC7A11, YAP, YAP phosphorylation and its targets expression in vector and MISP‐overexpressing H1395 cells. K) Examination of TEAD luciferase reporter activity in 293T cells upon MISP overexpression. L) Association of YAP with 14‐3‐3 by immunoblot analysis. 293T cells were co‐transfected with Myc‐14‐3‐3 and vector or Flag‐YAP in the presence of MISP. Co‐immunoprecipitates were detected using the indicated antibodies. M) Correlation analysis between MISP and the levels of CYR61 and CTGF in NSCLC patients (*n* = 40 (adjacent) and *n* = 40 (NSCLC)). Correlation was determined by the Pearson correlation test. Data are presented as mean ± SEM. One‐way ANOVA was used to compare differences between groups in C and E. Unpaired *t*‐test was used to determine statistical significance in I and K. *p < 0.05, **p < 0.01, ***p < 0.001, ****p < 0.0001.

To further validate this, we performed immunostaining to assess YAP localization in cells following MISP deletion. We observed a significant enhancement of YAP nuclear exclusion in *MISP*‐deficient H1395 cells (**Figure** **3**A,B), which was accompanied by SLC7A11 downregulation, increased YAP phosphorylation, and reduced YAP and CYR61 levels (Figure 3C,D). Using TEAD responsive luciferase reporter (8XGITTC), we observed impaired luciferase reporter activity in cells upon MISP knockdown (Figure 3E), further suggesting a positive regulation of MISP on YAP/TEAD signaling. Considering that YAP phosphorylation and cytoplasmic retention generally result in its ubiquitination and subsequent degradation in a 14‐3‐3‐dependent manner,^[^ ^9^ ^]^ we sought to determine whether MISP can protect YAP from degradation via 14‐3‐3. Consistent with our hypothesis, MISP ablation in cells led to increased interaction between YAP and 14‐3‐3 (Figure 3F), as well as enhanced YAP ubiquitination (Figure S3I, Supporting Information), suggesting that MISP indeed plays a key role in preventing YAP degradation through 14‐3‐3. Conversely, MISP overexpression promoted YAP nuclear retention (Figure 3G,H). The enhancement of YAP activation due to MISP overexpression is further supported by the increased expression of YAP target genes (Figure 3I,J; Figure S3J,K, Supporting Information). In addition, MISP overexpression augments TEAD luciferase reporter activity, a proxy for YAP‐TEAD complex formation and transcriptional output (Figure 3K). Concurrently, there is a dissociation of YAP from 14‐3‐3 proteins and reduced YAP ubiquitination, which is known to negatively regulate YAP by retaining it in the cytoplasm (Figure 3L; Figure S3L, Supporting Information). In clinical samples of lung cancer, a noteworthy positive correlation was observed among the expression levels of MISP, CYR61, and CTGF (Figure 3M). Collectively, these findings demonstrate that MISP functions as a novel regulator of the Hippo signaling pathway, stimulating the expression of SLC7A11.

### 2.4 MISP is a Target of Hippo‐YAP Signaling

**Figure 4:**
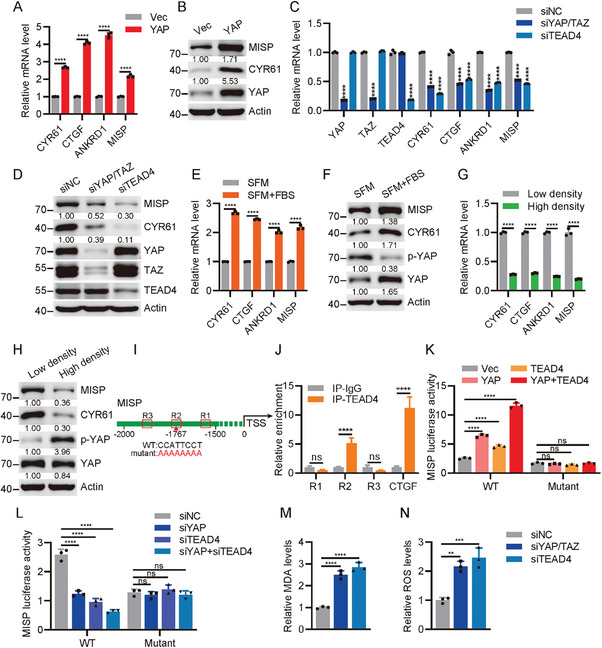
MISP is a target gene of YAP/TEAD signaling. A) Examination of MISP and YAP target genes in H1395 cells with or without YAP overexpression. B) Immunoblot analysis of MISP and CYR61 expression in vector and YAP‐overexpressing H1395 cells. C) Determination of MISP and YAP target genes in H1395 cells upon YAP/TAZ or TEAD4 depletion. D) Immunoblot analysis of MISP and CYR61 expression in H1395 cells upon YAP/TAZ or TEAD4 depletion. E,F) Determination of the mRNA E) and protein F) levels of MISP and YAP target genes in H1395 cells upon serum starvation or FBS recovery after serum starvation. H1395 cells were cultured in serum‐free medium (SFM) for 12 h and treated with FBS‐containing medium for 1 h. G,H) Determination of MISP and YAP target genes in H1395 cells at low or high cell density. H1395 cells were plated at different cell densities for 12 h and subjected to qPCR G) and immunoblotting H) analysis. I) Schematic diagram of the MISP promoter with conserved TEAD‐binding motifs (termed as R1, R2, and R3). TSS, transcription starting site. J) ChIP‐qPCR analysis of TEAD4 enrichment at the MISP and CTGF promoters. K) Analysis of luciferase reporter activity driven by the MISP promoter or R2‐defective MISP promoter in HEK293T cells transfected with the indicated plasmids. L) Analysis of luciferase reporter activity driven by the MISP promoter or R2‐defective MISP promoter in HEK293T cells transfected with the indicated siRNAs. M,N) Relative levels of MDA M) and ROS N) in H1395 cells upon YAP/TAZ or TEAD4 depletion. Data are presented as mean ± SEM. Unpaired *t*‐test was used to determine statistical significance in A, E, G, and J. One‐way ANOVA was used to compare differences between groups in C and K–N. *p < 0.05, **p < 0.01, ***p < 0.001, ****p < 0.0001.

Inspired by the concept that feedback loops are a common feature of Hippo signaling,^[^ ^25^ ^]^ we sought to test this hypothesis. Our results confirmed that overexpression of YAP significantly enhanced the expression of MISP along with its canonical targets, including CTGF, CYR61, and ANKRD1 in H1395 and HCC827 cells (**Figure** **4A,B**; Figure S4A,B, Supporting Information). In contrast, knockdown of YAP or TEADs by siRNA led to a substantial decrease in MISP and Hippo targets (Figure 4C,D; Figure S4C,D, Supporting Information). Given that the Hippo pathway senses various intrinsic and extrinsic cues, such as serum starvation and cell density, we observed that lung cancer cells, when subjected to serum deprivation followed by serum recovery, exhibited a marked increase in MISP and YAP targets (Figure 4E,F; Figure S4E,F, Supporting Information). Conversely, under high cell density conditions that activate Hippo signaling, the expression of MISP as well as YAP targets was reduced (Figure 4G,H; Figure S4G,H, Supporting Information). We then investigated whether MISP is a direct target of YAP/TEAD complex. Analysis of Chromatin immunoprecipitation followed by sequencing (ChIP–seq) datasets for TEAD4 revealed three conserved TEAD4‐binding motifs (termed as R1, R2, and R3) within the MISP promoter region (Figure 4I). ChIP‐qPCR assays confirmed TEAD4's binding to the second TEAD4‐binding region (R2) of MISP (Figure 4J). This suggests that MISP might be a TEAD4 target gene. The hypothesis was further validated through a MISP promoter luciferase reporter assay, which indicated that R2 mutation substantially diminished MISP promoter activity in cells overexpressing either YAP, TEAD4, or YAP/TEAD, as opposed to those with vector control (Figure 4K,L). Taken together, these data highlight a direct regulatory role of YAP/TEAD4 for MISP. Although previous studies have indicated a negative regulation of ferroptosis by YAP activation, we determined whether YAP activation suppresses ferroptosis in lung cancer cells. As expected, increased MDA and ROS production was observed in H1395 and PC9 cells (Figure 4M,N; Figure S4I–L, Supporting Information). Therefore, we reveal a MISP/YAP feedback loop implicated in ferroptosis.

### 2.5 MISP Binds to MST1/2 Kinases

**Figure 5:**
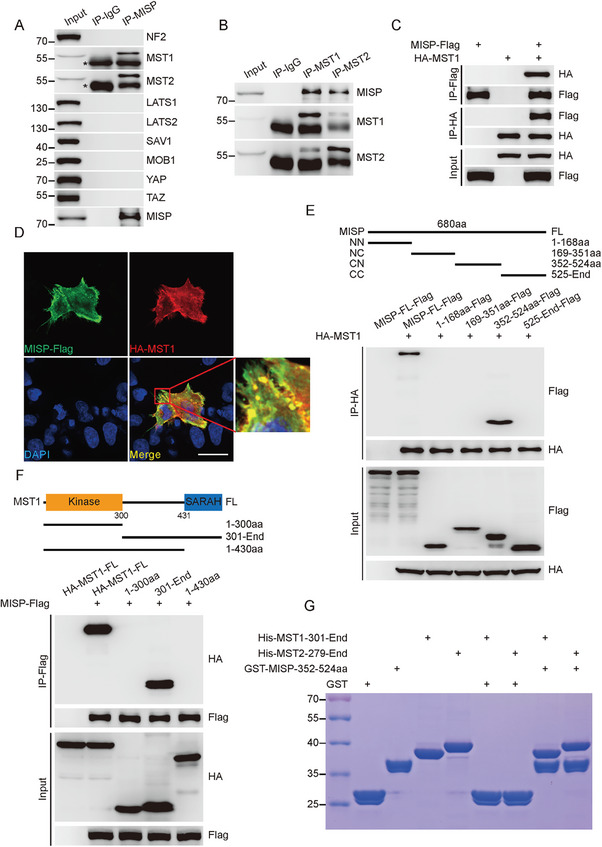
MISP interacts with MST1/2 kinases. A) Binding of MISP to MST1/2 kinases. PC‐9 cells were lysed and subjected to immunoprecipitation with IgG control or MISP antibody. Immunoprecipitates were detected using indicated antibodies. The IgG heavy chain is denoted by an asterisk. B) Interaction between MISP and MST1/2. Immunoprecipitation was performed with IgG control, MST1, or MST2 antibodies, and the interaction between MISP and MST1/2 was detected. C) MISP binds to MST1 in 293T cells. 293T cells were transfected with MISP‐Flag and HA‐MST1 plasmids alone or in combination for 24 h, lysed, and subjected to immunoprecipitation using the indicated antibodies. D) Immunofluorescent staining of MST1 and MISP using the indicated antibodies in H1395 cells. The boxed inset within the left‐hand panels highlights the specific area depicted in the enlarged image (right panel). Scale bar, 40 µm. E) The aa352‐524 region of MISP is required for MISP/MST1 interaction. Diagram of the domains and structural motifs of MISP (upper). Immunoblotting analysis of MISP fragments using the indicated antibody after Co‐IP with anti‐HA tag antibody (lower). F) The SARAH domain is required for MISP/MST1 interaction. Diagram of the domains and structural motifs of MST1 (upper). Immunoblot analysis of MST1 fragments using the indicated antibody after Co‐IP with anti‐Flag tag antibody (lower). G) Coomassie brilliant blue staining after GST or GST‐MISP pulldown. His‐MST1 (aa301‐487) or His‐MST2 (aa279‐491) recombinant proteins were incubated with GST or GST‐MISP (aa352‐524), and detected by Coomassie brilliant blue staining. Ten percent of each recombinant protein was used as input.

To clarify the detailed mechanisms by which MISP regulates the Hippo signaling pathway, co‐immunoprecipitation (Co‐IP) and liquid chromatography‐mass spectrometry (LC‐MS) were used to identify potential MISP binding partners within this cascade. Notably, MST1 was identified as one such candidate (Figure S5A, Supporting Information). We have confirmed the interaction between MISP and MST1/2 in PC‐9 cells immunoprecipitated using MISP or MST1/2 antibodies, respectively, and also in 293T cells exogenously (**Figure** **5**A–C; Figure S5B, Supporting Information). Notably, MISP and MST1/2 showed strong membrane colocalization, as determined by immunostaining (Figure 5D; Figure S5C, Supporting Information). To further identify specific domains responsible for this interaction, constructs encoding various regions of MISP and MST1/2 were prepared for Co‐IP analysis. The analysis revealed that amino acids spanning 352–524 (aa352‐524) of MISP are essential for its interaction with MST1/2 (Figure 5E; Figure S5D, Supporting Information), and aa431‐487, the C‐terminal SARAH domain, mediates MST1/2 binding to MISP (Figure 5F; Figure S5E, Supporting Information). This finding was further supported by a GST pulldown assay, which indicated a direct interaction between aa352–524 of MISP and the SARAH domain of MST1/2 (Figure 5G). Together, these data provide evidence for a new MISP‐MST1/2 axis for regulating the Hippo signaling pathway.

### 2.6 MISP Impairs MST1/2 Homo‐Dimerization and Activation

**Figure 6:**
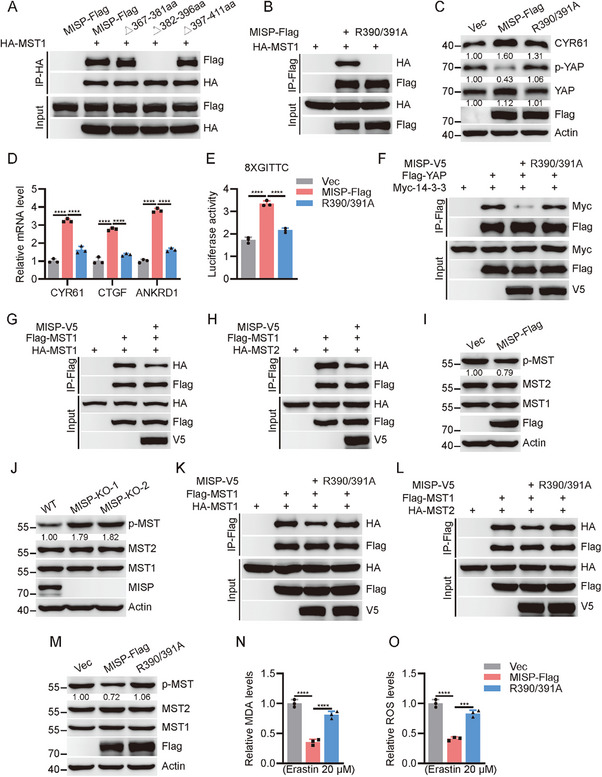
R390 and R391 of MISP are essential for interaction with MST1/2 kinases. A) Identification of the aa382‐396 region required for MISP/MST1 interaction. Indicated deletion mutants were co‐transfected with MST1 and subjected to immunoprecipitation. B) Determination of the binding affinity between WT MISP and the R2A mutant (R390/391A) for MST1. C) Immunoblot analysis of YAP phosphorylation, YAP, and CYR61 expression in H1395 cells in the presence of vector, WT MISP, or R2A mutant. D) Determination of YAP target genes expression in H1395 cells in the presence of vector, WT MISP, or R2A mutant. E) Examination of TEAD luciferase reporter activity in 293T cells upon forced expression of vector, WT MISP, or R2A mutant. F) Effects of WT MISP and R2A mutant on the association of YAP with 14‐3‐3 by Co‐IP and immunoblot analysis. G) Determination of MST1 homodimerization by Co‐IP and immunoblot analysis in 293T cells upon forced expression of vector, or WT MISP. H) Determination of MST1/2 heterodimerization by Co‐IP and immunoblot analysis in 293T cells upon forced expression of vector, or WT MISP. I) Immunoblot analysis of MST1 phosphorylation in H1395 cells upon MISP expression. J) Immunoblot analysis of MST1 phosphorylation in WT and *MISP*‐deficient H1395 cells. (K‐L) Effects of WT MISP and R2A mutant on MST1 homodimerization K) and heterodimerization L) by Co‐IP and immunoblot analysis in 293T cells. M) Immunoblot analysis of MST1 phosphorylation in H1395 cells upon WT MISP and R2A mutant expression. N,O) Relative levels of MDA N) and ROS O) in H1395 cells upon WT MISP and R2A mutant expression treated with Erastin (20 µM) for 24 h. Data are presented as mean ± SEM. One‐way ANOVA was used to compare differences between groups. *p < 0.05, **p < 0.01, ***p < 0.001, ****p < 0.0001.

To identify the key minimal amino acids necessary for the interaction between MISP and MST1/2, we generated deletion mutants in the amino acid sequence 352–524 of MISP. Co‐IP analysis showed that the minimal aa382–396 are indispensable for their association (**Figure** **6**A; Figure S6A, Supporting Information). Utilizing molecular docking with AlphaFold 3, we identified two critical arginine residues, R390 and R391, at the interface of the interaction. Indeed, replacement of R390 and R391 with alanine (R2A) completely abolished its binding to MST1 and MST2 (Figure 6B; Figure S6B, Supporting Information). The R2A mutant had defects in inducing YAP activation and stimulating the expression of target genes in H1395 and HCC827 cells (Figure 6C,D; Figure S6C,D, Supporting Information). Moreover, cells expressing the R2A mutation exhibited decreased YAP nuclear translocation and TEAD luciferase reporter activity when compared to cells with wild‐type MISP (Figure 6E; Figure S6E, Supporting Information). Given that YAP phosphorylation typically resulted in association with 14‐3‐3 and ubiquitination‐dependent degradation, we then tested whether the MST1/2 binding‐defective R2A mutant alters the above effect. Indeed, YAP interaction with 14‐3‐3 and ubiquitination were greatly enhanced in cells with the R2A mutant compared with wild‐type MISP (Figure 6F; Figure S6F, Supporting Information). These data indicated an essential role of R390 and R391 in MISP for its interaction with MST1/2 and subsequent YAP activation. Since the SARAH domain is critical for the homodimerization of the MST1/2 kinases and subsequent autophosphorylation, we sought to determine whether the SARAH domain in association with MISP affects the homodimerization and activation of MST1/2 kinases. To this end, we found that the addition of MISP significantly prevented MST1/2 interaction with themselves (Figure 6G,H; Figure S6G, Supporting Information), and inhibited MST kinases phosphorylation (Figure 6I; Figure S6H, Supporting Information). Conversely, MST1/2 phosphorylation was remarkably enhanced in MISP‐ablated cells (Figure 6J; Figure S6I, Supporting Information), indicating that MISP indeed modulates MST1/2 kinases dimerization and autophosphorylation. Furthermore, unlike MISP, the R2A mutant, which disrupts MISP's binding to MST1/2 kinases, failed to restrain MST1/2 kinases dimerization and autophosphorylation (Figure 6K–M; Figure S6J,K, Supporting Information). Next, we test whether the binding‐defective mutant affects its role in ferroptosis inhibition. Indeed, R2A mutant showed remarkably reduced capability to inhibit MDA and ROS production, and impaired SLC7A11 expression, in contrast to MISP wild type (Figure 6N,O; Figure S6L–O, Supporting Information), indicating that MISP suppresses ferroptosis that is dependent on its binding to MST kinases and subsequent activation. Overall, these results demonstrate that MISP interacts with MST1/2 kinases to limit MST1/2 homodimerization and activation.

### 2.7 MISP Triggers YAP Activation and Tumor Progression via MST1/2 Kinases

**Figure 7:**
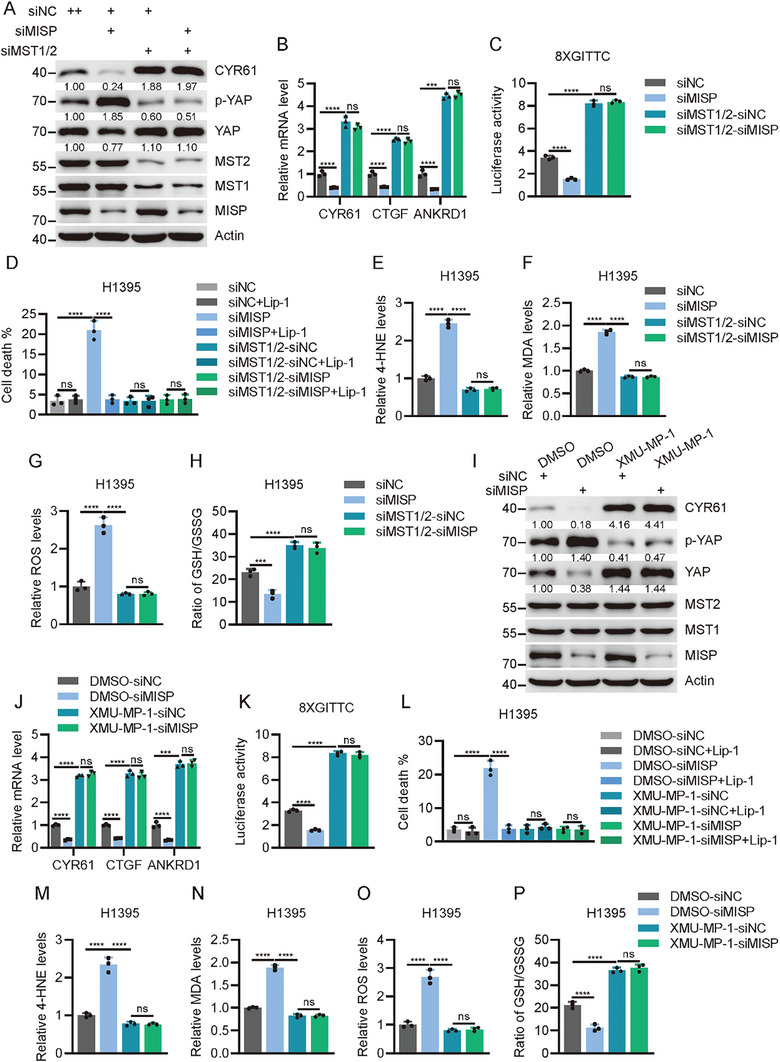
MST1/2 kinases are required for MISP to trigger YAP activation. A) Immunoblot analysis of YAP phosphorylation, YAP, and CYR61 expression in H1395 cells upon transfection with the indicated siRNA. B) Determination of YAP target genes by qPCR in H1395 cells upon transfection with the indicated siRNA. C) Evaluation of TEAD luciferase reporter activity in 293T cells upon transfection with the indicated siRNA. D) Determination of cell death by trypan blue staining in cells with indicated treatment. E–H) Relative levels of 4‐HNE E), MDA F), ROS G), and GSH/GSSG H) in H1395 cells upon transfection with the indicated siRNA. I) Immunoblot analysis of YAP phosphorylation, YAP, and CYR61 expression in H1395 cells upon transfection with the indicated siRNA with or without XMU‐MP‐1 treatment (2.5 µM for 12 h). J) Determination of YAP target genes by qPCR in H1395 cells following transfection with the indicated siRNA with or without XMU‐MP‐1 treatment (2.5 µM for 12 h). K) Determination of TEAD luciferase reporter activity in 293T cells upon transfection with the indicated siRNA with or without XMU‐MP‐1 treatment (2.5 µM for 12 h). L) Determination of cell death by trypan blue staining in H1395 cells with indicated treatment. M–P) Relative levels of 4‐HNE M), MDA N), ROS O), and GSH/GSSG P) in H1395 cells upon transfection with the indicated siRNA with or without XMU‐MP‐1 treatment (2.5 µM for 12 h). Data are presented as mean ± SEM. One‐way ANOVA was used to compare differences between groups. *p < 0.05, **p < 0.01, ***p < 0.001, ****p < 0.0001.

**Figure 8:**
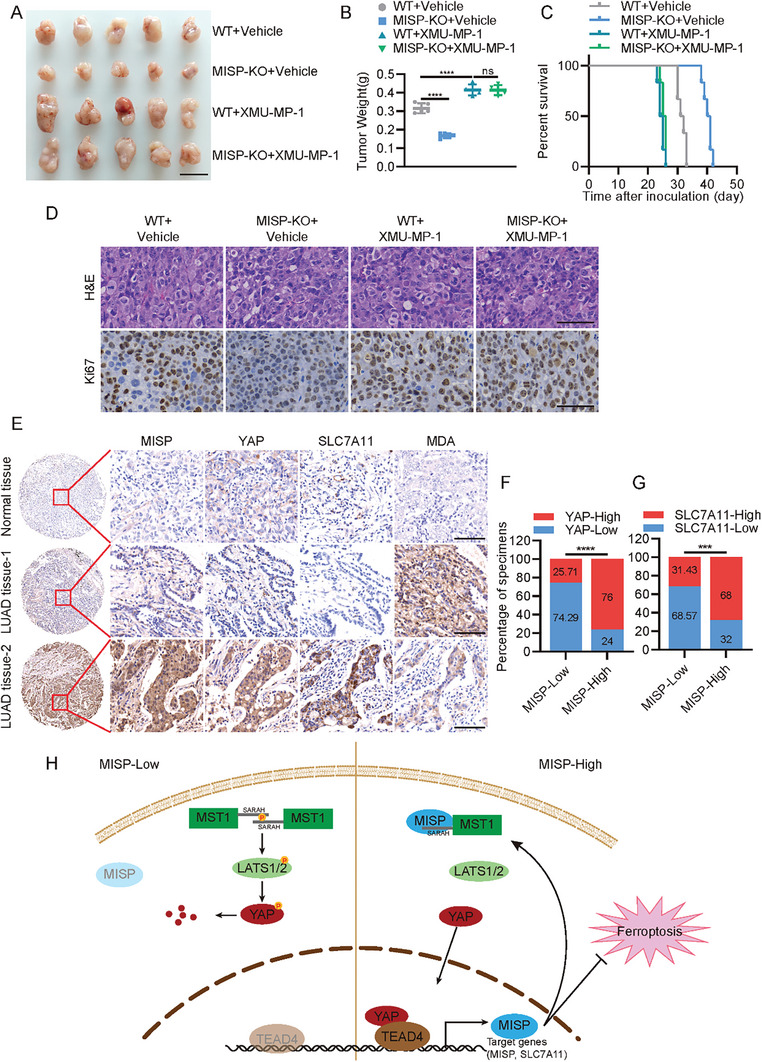
MISP promotes tumor growth in vivo and correlates with YAP and SLC7A11 in NSCLC samples. A) Gross images of xenografts from the indicated mice with or without XMU‐MP‐1 treatment (5 mg kg^−1^). Scale bar, 10 mm. B) Analysis of tumor weight from the indicated groups. C) Kaplan‐Meier plots showing the overall survival of the indicated nude mice with or without XMU‐MP‐1 treatment (5 mg kg^−1^). D) Representative images of H&E and IHC staining of Ki67 in the indicated tumor sections. Scale bar, 50 µm. E) Representative images of IHC staining of MISP, YAP, SLC7A11, and MDA in NSCLC specimens. Scale bars, 150 µm. F,G) Correlation analysis between MISP and the levels of YAP (F) and SLC7A11 G) in NSCLC samples. Samples with grades 0 (no staining) and 1 (weak staining) were grouped as “low” expressions and samples with grades 2 (strong staining) were grouped as “high” expressions. H) Proposed working model describing the MISP‐MST1/2‐SLC7A11 signaling axis dictating ferroptosis and lung cancer growth. Data are presented as mean ± SEM. One‐way ANOVA was used to compare differences between groups. *p < 0.05, **p < 0.01, ***p < 0.001, ****p < 0.0001.

Given the essential role of this interaction in blunting MST1/2 homodimerization and activation, we sought to explore whether MST1/2 kinases are required for MISP‐mediated YAP activation. In agreement with the above results, MISP knockdown in cells led to YAP phosphorylation, repression of its target gene expression, and diminished TEAD‐responsive promoter activity, which was not observed in MST1/2‐deficient cells (**Figure** **7**A–C; Figure S7A,B, Supporting Information). Similarly, deficiency of MST1/2 kinases restored cell death, lipid peroxidation, and ferroptosis caused by MISP depletion in lung cancer cells (Figure 7D–H; Figure S7C–G, Supporting Information). To further support this observation, we extended our analysis to include XMU‐MP‐1, a small inhibitor of MST1/2 kinases. Our analysis indicated that H1395 and PC‐9 cells after XMU‐MP‐1 treatment showed largely rescued YAP activation as well as ferroptosis induced by MISP loss (Figure 7I–P; Figure S7H–N, Supporting Information). We next tested the in vivo effects of the MISP‐MST1/2 signaling axis in nude mice treated with or without XMU‐MP‐1. As expected, smaller tumors were seen in *MISP*‐deficient H1395 cells relative to WT cells, a difference that was mitigated in MISP‐depleted cells following XMU‐MP‐1 treatment (**Figure** **8**A,B). Similarly, other processes affected by this include overall survival times and Ki‐67 reactivity (Figure 8C,D). To provide clinical relevance, we next analyzed the expression of MISP, YAP, and SLC7A11 in NSCLC specimens. Indeed, a positive correlation between MISP expression and the levels of YAP and SLC7A11 was observed in NSCLC samples (Figure 8E–G). Taken together, the data here demonstrate that MST1/2 kinases are indispensable for MISP to activate YAP and promote tumor growth in lung cancer (Figure 8H). This underscores the significance of MST1/2 kinases in the MISP‐mediated regulation of YAP and highlights their potential as therapeutic targets in lung cancer treatment.

## 3 Discussion

Ferroptosis, an iron‐dependent form of cell death characterized by lipid peroxidation, has emerged as a significant player in cancer biology.^[^ ^17^, ^20^, ^21^ ^]^. In NSCLC, ferroptosis inducers such as erastin have shown potential in treating the disease, especially in combination with inhibitors of the NRF2 pathway.^[^ ^26^ ^]^ The role of ferroptosis in modulating responses to traditional chemotherapy and radiotherapy in NSCLC is gaining attention, with evidence suggesting that inducing ferroptosis can overcome drug resistance.^[^ ^27^, ^28^ ^]^ The molecular machinery of ferroptosis, including the regulation of key enzymes like GPX4 and ACSL4, is being targeted to enhance the effectiveness of cancer treatments and to tackle resistant tumors, highlighting ferroptosis as a critical area of focus for novel cancer therapeutics.^[^ ^29^, ^30^ ^]^ Analysis in various datasets revealed MISP as a novel oncogene related to ferroptosis, and MISP is hence selected for further study. Our study identified indeed MISP as a pivotal inhibitor of ferroptosis in NSCLC. MISP upregulation is associated with resistance to ferroptosis, which could be a mechanism of therapeutic resistance in NSCLC. Our work also supports the emerging view that modulating ferroptosis could be a viable therapeutic strategy. Targeting MISP could sensitize NSCLC cells to ferroptosis, potentially offering a new avenue for overcoming resistance.

While the Hippo pathway is well‐established in controlling cell proliferation and organ size, it has recently emerged as a regulator of ferroptosis in cancers. YAP/TAZ have been reported to confer resistance to ferroptosis in hepatocellular carcinoma.^[^ ^18^, ^31^ ^]^ Conversely, TAZ has been shown to increase cellular sensitivity to ferroptosis by upregulating epithelial membrane protein 1, which in turn induces the expression of NADPH oxidase 4, a key player in ferroptosis.^[^ ^32^, ^33^ ^]^ In addition, the NF2‐YAP signaling axis has been implicated in regulating ferroptotic death in cancer cells, with genetic inactivation of the tumor suppressor NF2 rendering cancer cells more sensitive to ferroptosis.^[^ ^17^ ^]^ Here, we demonstrated that SLC7A11 is a target of YAP and revealed a positive regulation of YAP on SLC7A11 by MISP, suggesting that the Hippo pathway could be a double‐edged sword in cancer progression and treatment,^[^ ^34^ ^]^ which might depend on cell‐type‐specific regulation mechanisms. We further identified MISP as a bona fide target of YAP, creating a positive feedback loop that amplifies YAP signaling, reinforcing the notion that the dynamic feedback loop is a fundamental principle to mitigate signal fluctuation commonly observed in the Hippo pathway.^[^ ^25^ ^]^

The role of MST1/2 as a tumor suppressor is well‐established, with loss of function leading to cell hyperproliferation and tumorigenesis. However, the precise mechanisms by which MST1/2 activity is regulated in cancers remain unclear. MST1/2 kinases contain C‐terminal SARAH domains that mediate their homodimerization and facilitate autophosphorylation and activation.^[^ ^35^ ^]^ Moreover, MST1 and MST2 also heterodimerize in cells, leading to MST1/2 inactivation, depending on the order of RASSF5 binding and activation‐loop phosphorylation.^[^ ^36^, ^37^ ^]^ The observation that MISP disrupts both MST1/2 homodimerization and heterodimerization here provides one possible explanation for MST1/2 inactivation in cancers with aberrant MISP, at least in NSCLC. It is not clear how MISP integrates diverse signals to MST1/2 and/or possibly RASSF5, which is an interesting topic for future work. However, the mechanistic link between MISP and MST1/2 kinases provides a basis for expanding the potential therapeutic applications of targeting MST1/2 via MISP in the Hippo signaling pathway, in contrast to conventional strategies that target downstream effectors such as YAP and TEADs.

Notably, MISP contains multiple actin‐binding sites and is implicated in cellular cytoskeletal reorganization, which is established as a player in the regulation of the Hippo pathway, mediated by filamentous actin (F‐actin) accumulation.^[^ ^6^, ^8^ ^]^ It is not clear whether MISP also contributes to Hippo signaling via F‐actin and cytoskeletal remodeling in other tumors, and it is an interesting work warranting future investigation. Notably, although we confirmed that MISP is linked to ferroptosis via Hippo signaling, additional signaling pathways might be potentially involved.

While our study adds insights into the Hippo signaling and ferroptosis in cancer, it has limitations. The mechanistic understanding of MISP's role in ferroptosis requires further validation across different NSCLC subtypes and various cancer types as to whether MISP‐MST1/2 signaling axis represents a common mechanism in remodeling ferroptosis in cancers. Also, we cannot rule out potentially off‐target effects in vitro assays. Future works should be performed using Genetically engineered mouse models (GEMMs), which develop *de novo* tumors in a natural immune‐proficient microenvironment. Given that targeting ferroptosis is a potential strategy to enhance targeted or immunotherapy, it is not clear whether MISP is implicated in regulation of lung cancer immune microenvironment, which should be an interesting work in the future. Additionally, the translational potential of targeting MISP (e.g., developing small inhibitors) for therapeutic purposes needs to be further explored in preclinical models. Collectively, our study uncovers the critical role of MISP in the regulation of ferroptosis in NSCLC, highlighting a potential therapeutic target. The findings suggest that modulating the MISP‐YAP‐SLC7A11 axis could overcome therapeutic resistance and improve outcomes for NSCLC patients.

## 4 Experimental Section

### Sample Collection

A total of 85 non‐small cell lung cancer (NSCLC) tissue microarrays (TMA) were collected from the Second Affiliated Hospital of Nantong University. This collection was conducted with the full understanding and written consent of each participant involved. All procedures were performed under the approval of the Ethics Committee of the Second Affiliated Hospital of Nantong University (approval no. 2022KT246).

### Animal Studies

One million (1 × 10^6^) wild‐type (WT) H1395 or MISP‐depleted H1395 cells, suspended in 100 µL of phosphate‐buffered saline (PBS), were subcutaneously inoculated into the left flank of five‐week‐old male BALB/c nude mice (*n* = 5 per group). To assess drug response, the mice were administered either vehicle control or the drug XMU‐MP‐1 (5 mg kg^−1^) via intraperitoneal injections every three days for three weeks, starting when the tumors reached a volume of 224850 mm^3^. At the end of the experiment, the tumors were harvested and their weights were measured and examined. For survival analysis, the mice were monitored daily for signs of disease, which determined the endpoint for euthanasia, and the survival times were subsequently analyzed. All experimental procedures were conducted with the approval of the Research Ethics Committee of Nantong University (approval no. S20220221‐040).

### Cell culture, Cell proliferation, and Colony Formation Assay

Human Embryonic Kidney (HEK) 293T, PC‐9, NCI‐H1395, NCI‐H1975, and HCC827 cell lines were purchased from the Cell Bank of Shanghai Institutes of Biological Sciences and maintained in DMEM medium or RPMI‐1640 medium (Gibco) supplemented with 10% FBS at 37 °C in a 5% CO2 incubator. BEAS‐2B and 16HBE cell lines were obtained from ATCC and maintained in a special culture medium (CM‐0496 and CM‐0249, Procell). Cell proliferation in non‐small cell lung cancer (NSCLC) cells was assessed using the CCK‐8 assay. Each experiment was conducted in triplicate, with each group being tested in quintuplicate to ensure statistical reliability. For the colony formation assay, cells were infected with the indicated lentivirus and then cultured in suspension medium. A total of 300–500 cells infected with the indicated lentivirus were transferred into 6‐well plates and incubated for 2–3 weeks to allow colony formation. After incubation, the plates were gently washed twice with phosphate‐buffered saline (PBS) to remove any debris, followed by fixation with 4% paraformaldehyde for 20 min. The number of colonies was counted and statistically analyzed to assess the cell proliferation and colony formation capabilities.

### RNA Interference and Knockout Cell Preparation

MISP, MST1, MST2, YAP/TAZ, and TEAD4 Knockdown were achieved by RNA interference using short interfering RNA (siRNA) and lentiviral vector‐based shRNA. The siRNA and shRNA sequences used are listed in Tables S1 and S2 (Supporting Information). shRNA lentivirus particles were prepared as previously described.^[^ ^38^, ^39^ ^]^ MISP knockout in H1395 cells was generated using the CRISPR–Cas9 system. Briefly, two single‐guide RNAs (sgRNAs) were designed to target the MISP gene. These sgRNAs were then constructed into the lenti‐CRISPR V2 vector. H1395 cells were infected with lentiviruses carrying sgMISP‐1 and sgMISP‐2 for 24 h, followed by selection with puromycin treatment for an additional 24 h. After selection, individual puromycin‐resistant cells were isolated and plated into 96‐well plates for clonal expansion over 2–3 weeks. Positive clones were identified and validated by immunoblotting and DNA sequencing to confirm successful knockout of the MISP gene. The sgRNA sequences are listed in Table S3 (Supporting Information).

### Reagents and Antibodies

Necrostatin‐1 (S8037), Z‐VAD‐FMK (S7023), 3‐Methyladenine (S2767), Erastin (S7242), and XMU‐MP‐1 (S8334) were purchased from Selleck (Shanghai, China). Fraction of nuclear and cytoplasmic proteins was prepared using NE‐PER Nuclear and Cytoplasmic Extraction Reagents (78 833, Thermo Scientific). Antibodies used for immunoblot and Immunoprecipitation are listed below. MST1 (1:1000, 14 946), MST2 (1:1000, 3952), Phospho‐YAP (1:1000, 13 008), and Phospho‐MST1 (1:1000, 49 332) were purchased from Cell Signaling Technology. MISP (1:1000, 26338‐1‐AP), CYR61 (1:1000, 26689‐1‐AP), SLC7A11(1:1000, 26864‐1‐AP) were obtained from ProteinTech (Wuhan, China); Rabbit‐IgG (1:1000, sc‐2027) and Mouse‐IgG (1:1000, sc‐2025) were purchased from Santa Cruz Biotechnology. YAP (1:1000, WH0010413M1), Flag (1:1000∼5000, F9291), HA (1:1000∼5000, H9658) and V5 (1:1000, H9658) antibodies were purchased from Sigma‐Aldrich. Tubulin (1:1000, AC015), Lamin A/C (1:10 000, A19524), Actin (1:50 000, AC026), and GAPDH (1:10 000, AC033) were purchased from Abclonal (Wuhan, China).

### Immunoprecipitation and GST Pulldown

To isolate MISP‐interacting proteins, PC‐9 cells were infected with either MISP‐Flag‐tagged or control vector lentivirus. After infection, the cells were lysed to achieve a homogeneous mixture. The supernatant was incubated overnight with 20 µL of FLAG‐M2 agarose beads on a gentle rotator at 4 °C. Following incubation, the agarose beads were collected by centrifugation, and the pellet was washed five times with RIPA buffer, which contained 50 mm Tris (pH 7.4), 250 mm NaCl, 1% Triton X‐100, 1% sodium deoxycholate, 1 mm EDTA, and 20% glycerol. The immunoprecipitated proteins were then eluted by boiling in loading buffer and resolved by sodium dodecyl sulfate‐polyacrylamide gel electrophoresis (SDS‐PAGE). The proteins were visualized by Coomassie blue staining. Subsequent steps, including excision of protein bands, in‐gel trypsin digestion, peptide extraction, and liquid chromatography‐tandem mass spectrometry (LC‐MS/MS) analysis, were performed as previously described for protein identification.^[^ ^38^, ^39^ ^]^ For the GST pulldown assay, recombinant GST‐MISP (amino acids 352–524), His‐MST1 (amino acids 301–487), and His‐MST2 (amino acids 279–491) were expressed in *E. coli* BL21 and purified using glutathione S‐transferase (GST) and nickel‐nitrilotriacetic acid (Ni‐NTA) agarose, respectively. Equal amounts (2 µg) of GST or GST‐MISP recombinant protein were incubated with 2 µg of His‐MST1 or His‐MST2 for 12 hours at 4 °C. The resulting complexes were then eluted by boiling in loading buffer and analyzed by SDS‐PAGE and Coomassie blue staining.

### Real‐Time PCR and Luciferase Reporter Assay

Total RNA was isolated from cultured cells using TRIzol reagent (Invitrogen). cDNA was synthesized by reverse transcription and subjected to real‐time PCR with gene‐specific primers. The primer sequences are listed in Table S4 (Supporting Information). A total of 0.5 µg of the 8XGITTC luciferase reporter plasmid was co‐transfected with a Renilla vector into 293T cells with control or indicated plasmids for 24 h. For the pGL3‐MISP‐promoter construct, the 5′ flanking sequence of the human MISP gene was amplified by PCR from −2000 to −100 bp relative to the transcriptional start site. 293T cells were seeded in 24‐well plates, and either pGL3‐MISP‐promoter or the indicated mutant, along with pRL‐Renilla, were co‐transfected. 48 h after transfection, cells were lysed and luciferase activity was measured using the enhanced luciferase assay kit (Promega), following the manufacturer's instructions.

### Chromatin Immunoprecipitation‐PCR (ChIP‐PCR)

ChIP‐PCR was performed as previously described. Briefly, cells were crosslinked with 1% formaldehyde directly in culture medium for 10 min at room temperature. The collected samples were then sonicated to shear chromatin to an average fragment size of 200–600 bp. The chromatin fragments were immunoprecipitated with antibodies against TEAD4 (58 310; Abcam) or normal mouse IgG (sc‐2025; Santa Cruz) overnight. Antibody/antigen complexes were recovered with ProteinA‐Dynabeads (Invitrogen) for 2 h at 4 °C. The immunoprecipitated DNA was purified using the QIAquick PCR Purification Kit (QIAGEN). Purified DNA was then subjected to ChIP‐PCR. The amount of immunoprecipitated DNA in each sample was determined as the fraction of the input DNA and normalized to the IgG control. The primer sequences used are listed in Table S5 (Supporting Information).

### Detection of Malondialdehyde (MDA) and 4‐Hydroxynonenal (4‐HNE)

Lipid peroxidation was assessed using commercially available kits (Abcam, ab118970, and ab238538) to measure the levels of malondialdehyde (MDA) and 4‐hydroxy‐2‐nonenal (4‐HNE) in cell lysates, following the manufacturer's instructions. For MDA measurement, 1 × 10^6^ cells were harvested using MDA lysis buffer containing 1% butylated hydroxytoluene (BHT). After centrifugation, 200 µL of the supernatant was added to a 1.5 mL Eppendorf tube containing 600 µL of Developer VII/TBA reagent and incubated at 95 °C for 60 min. The reaction mixture was then cooled on ice for 10 min. Following this, 200 µL of the reaction mixture was transferred to a 96‐well plate, and absorbance at 532 nm was measured. For 4‐HNE measurement, 1 × 10^6^ cells were lysed with RIPA buffer, followed by centrifugation. Subsequently, 50 µL of the supernatant was added to a 4‐HNE conjugate‐coated plate and incubated for 10 min. After incubation with anti‐4‐HNE primary antibody and secondary antibody‐HRP, substrate solution was added and the absorbance at 450 nm was measured.

### Measurement of ROS Levels

The ROS Assay Kit (S0033, Beyotime) was used to determine the levels of ROS in cells. Briefly, cells were seeded in six‐well plates at a density of 3 × 10^5^ cells per well. The next day, cells were harvested and incubated with 10 µm DCFH‐DA at 37 °C for 30 min in the dark. After washing three times, ROS fluorescence intensity was assessed by flow cytometry.

### Immunofluorescent, Hematoxylin, and Eosin (H&E) and Immunohistochemical (IHC) Staining

For immunofluorescent staining, cells were fixed in 4% paraformaldehyde for 30 min, washed with PBS, permeabilized with 0.1% Triton X‐100 for 30 min, and blocked with 3% BSA in PBS for 30 min. The cells were then incubated with the primary antibody against YAP (1:1000 dilution, Sigma, catalog number WH0010413M1) overnight at 4 °C, followed by incubation with secondary antibodies (Jackson ImmunoResearch). Nuclei were counterstained with DAPI (40728ES03, YEASEN). Fluorescent images were captured using a Leica TCS SP8 confocal microscope. For histological analysis, paraffin‐embedded tumor tissues were stained with H&E as reported elsewhere. IHC staining was performed as previously reported.^[^ ^39^, ^40^ ^]^ The antibodies used for IHC were specific to MISP (1:200 dilution, catalog number 26338‐1‐AP), YAP (1:500 dilution, catalog number WH0010413M1), and SLC7A11 (1:200 dilution, catalog number 26864‐1‐AP). IHC staining was evaluated independently by two experienced pathologists. The staining intensity was categorized on a scale of 0 to 2, where 0 indicated no staining, 1 indicated weak staining, and 2 indicated strong staining. The extent of staining was quantified on a scale from 0 to 4. To determine the overall staining score, the extent score was multiplied by the intensity score, yielding a composite score that ranges from 0 to 8. Samples were then classified into low expression (scores 0–4) or high expression (scores 6–8) based on this composite score.

### Data Source and Statistical Analysis

Raw gene expression datasets and sample information pertaining to lung cancers were acquired from the Cancer Genome Atlas Program (TCGA) or the GEO database (GSE32863). To assess the prognostic significance of gene expression, survival analysis was conducted using the Log‐rank test to compare the overall survival rates between groups that were stratified based on the median expression levels of the genes of interest. Gene Set Enrichment Analysis (GSEA) was utilized for pathway enrichment by using gene expression datasets (TPM units) between indicated groups stratified by median expression level of the indicated gene. Data are expressed as mean ± SEM. Two‐group comparisons were performed using unpaired Student's *t*‐tests, while one‐way ANOVA was used for multiple‐group comparisons. Differences were considered statistically significant at two‐tailed *p* < 0.05 (*p < 0.05; **p < 0.01; ***p < 0.001; ****p < 0.0001). Results represent three independent experiments unless stated otherwise. Correlations were assessed with Pearson's correlation test, and survival analysis was conducted using the log‐rank (Mantel‐Cox) test. All statistical analyses were performed using GraphPad Prism software (GraphPad Software Inc.).

## Conflict of Interest

The authors declare no conflict of interest.

## Supporting information



Supporting Information

Supporting Information

## Data Availability

The data that support the findings of this study are available from the corresponding author upon reasonable request.
