# Supplementary material for: MISP Suppresses Ferroptosis via MST1/2 Kinases to Facilitate YAP Activation in Non‐Small Cell Lung Cancer
Source: Adv Sci (Weinh). 2025 Feb 28;12(16):2415814. doi: 10.1002/advs.202415814 (PMC12021056; doi:10.1002/advs.202415814)
Supplement: Supplementary file 1 — Supporting Information [file ADVS-12-2415814-s001.docx]

**Supplemental Figure:**


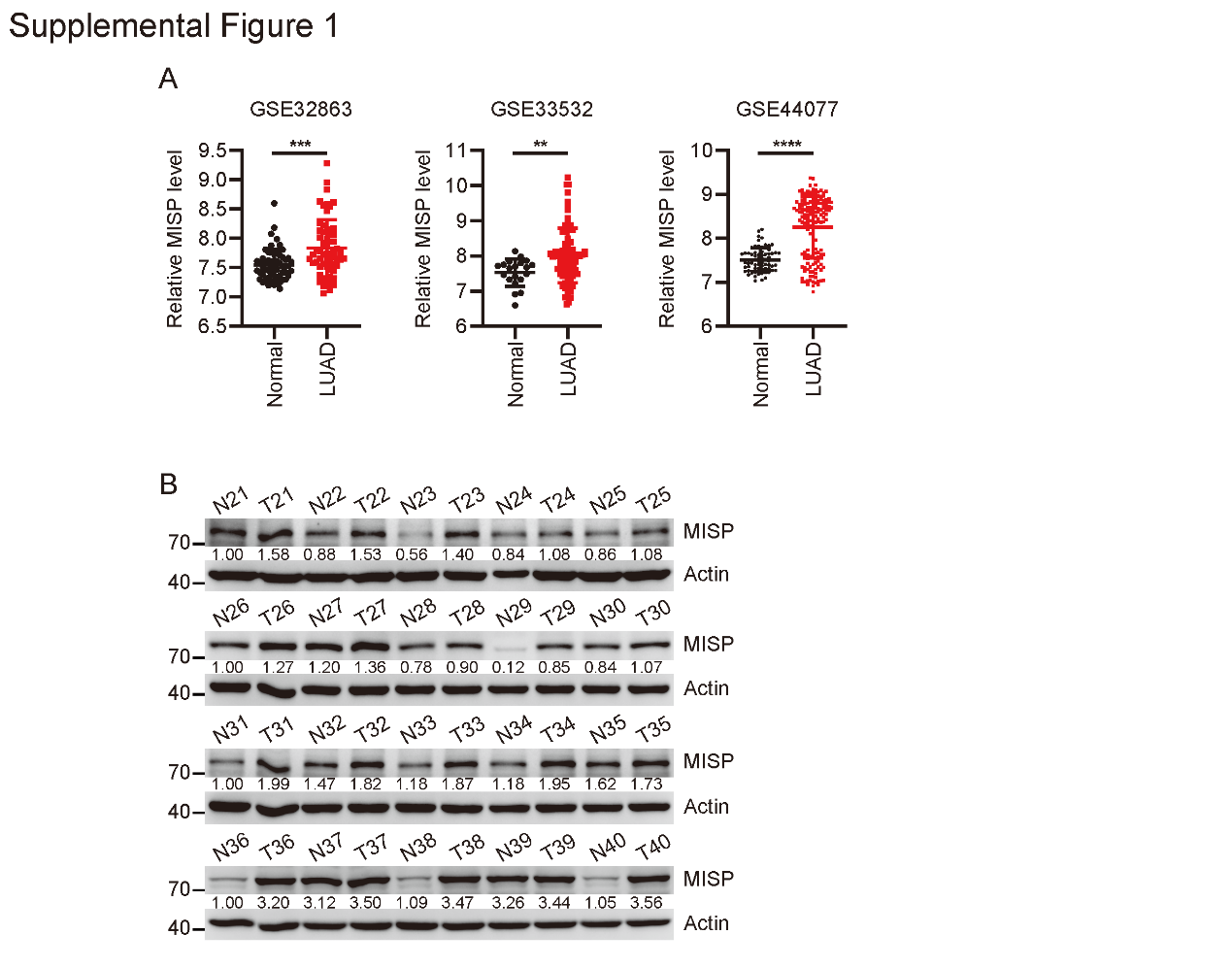


**Supplemental Figure 1: Upregulation of MISP in NSCLC.**

(A) Dot plot showing MISP expression in NSCLC and adjacent tissues from GEO dataset GSE32863 (n=58 (adjacent) and n=58 (NSCLC)), GSE33532 (n=20 (adjacent) and n=80 (NSCLC)) and GSE44077 (n=66 (adjacent) and n=160 (NSCLC)). (B) Immunoblot analysis of MISP expression in NSCLC and adjacent tissues (cases 21 to 40). n=20 (adjacent) and n=20 (NSCLC). Data are presented as mean ± SEM. Unpaired *t*-test was used in A to determine statistical significance. *, **, ***, **** indicate *p* < 0.05, *p* < 0.01, *p* < 0.001, *p* < 0.0001.


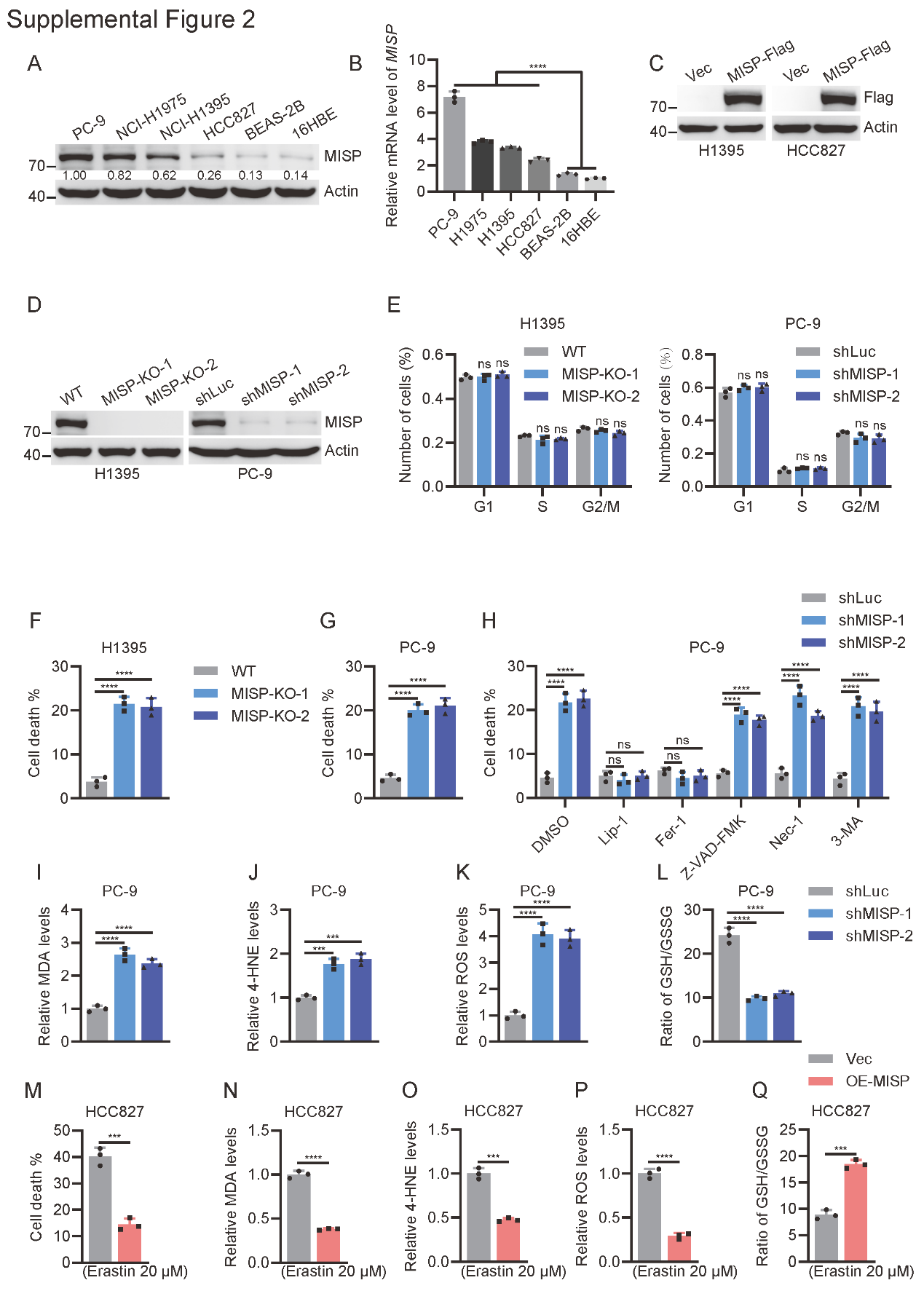


**Supplemental Figure 2: MISP suppresses ferroptosis in NSCLC cells.**

(A) Protein levels of MISP in a panel of NSCLC cell lines, human normal bronchial epithelial cells, and human normal lung epithelial cells were determined by Western blot. (B) Relative mRNA levels of MISP in a panel of NSCLC cell lines, human normal bronchial epithelial cells, and human normal lung epithelial cells were determined by qPCR. (C) Immunoblot verification of MISP overexpression in H1395 and HCC827 cells. (D) Immunoblot verification of MISP knockdown efficiency in H1395 and PC-9 cells. (E) Quantification of cell cycle distribution in indicated cells. (F-G) Trypan blue staining and quantification of cell death in WT and MISP-depleted H1395 cells (F) and PC-9 cells (G). (H) Trypan blue staining and quantification of cell death in WT and MISP-depleted PC-9 cells treated with DMSO, Lip-1 (500 nM), Fer-1 (10 μM), Z-VAD-FMK (10 μM), Nec-1 (10 μM) and 3-MA (1 mM) for 24 h. (I-L) Relative levels of MDA (I), 4-HNE (J), ROS (K) and GSH/GSSG (L) in WT and MISP-depleted PC-9 cells. (M) Trypan blue staining and quantification of cell death in vector and MISP-overexpressing HCC827 cells treated with Erastin (20 μM) for 24 h. (N-Q) Relative levels of MDA (N), 4-HNE (O), ROS (P) and GSH/GSSG (Q) in vector and MISP-overexpressing HCC827 cells treated with Erastin (20 μM) for 24 h. Data are presented as mean ± SEM. One-way ANOVA was used to compare differences between groups in B and E-L. Unpaired *t*-test was used to determine statistical significance in M-Q. *, **, ***, **** indicate *p* < 0.05, *p* < 0.01, *p* < 0.001, *p* < 0.0001.


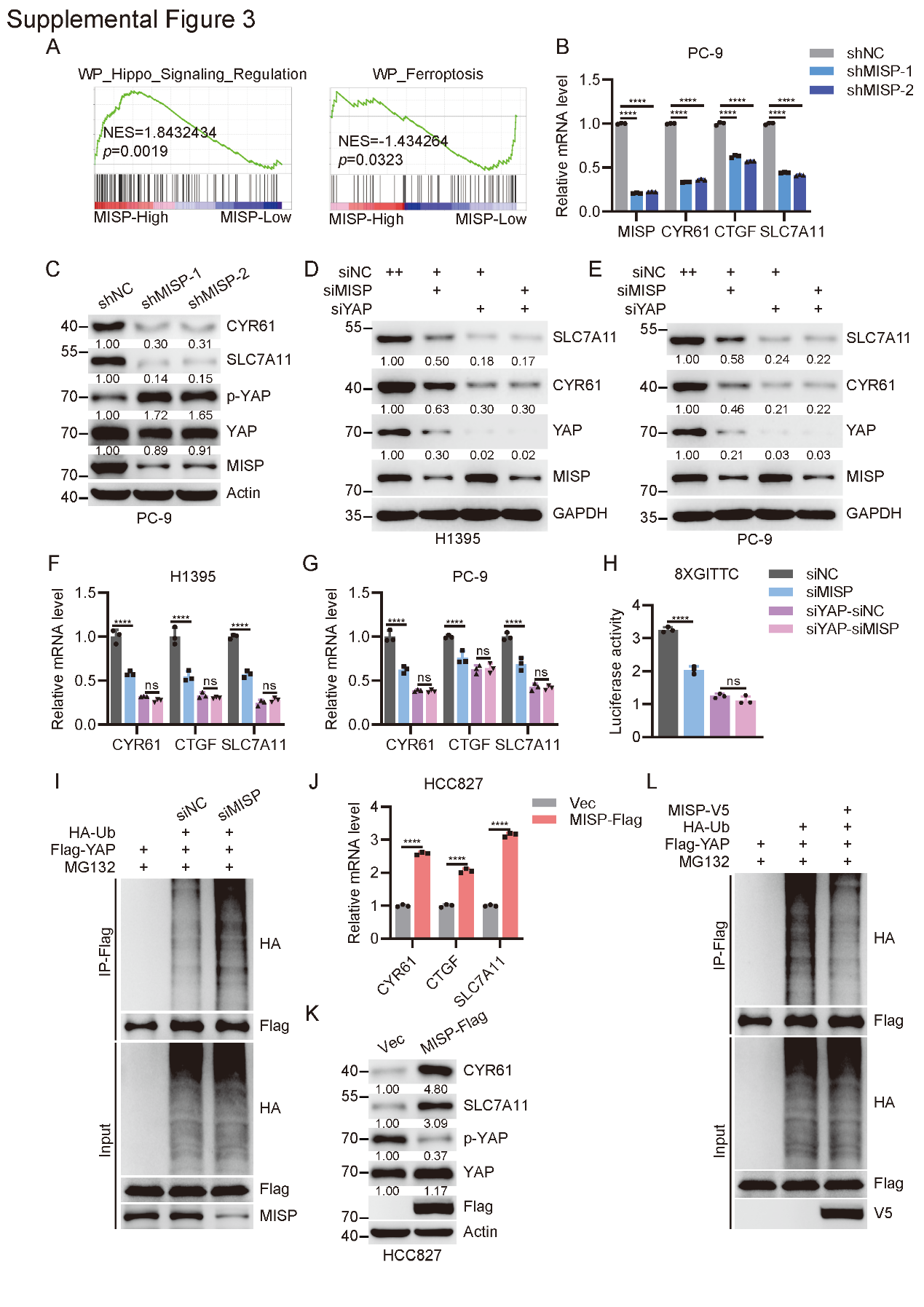


**Supplemental Figure 3: MISP suppresses Hippo signaling to trigger YAP activation and SLC7A11 expression.**

(A) Pathway enrichment using GSEA (Gene Set Enrichment Analysis) showed that higher MISP expression in NSCLC was associated with Hippo signaling and ferroptosis signature. (B) Measurement of SLC7A11 and YAP target gene levels in PC-9 cells upon MISP depletion. (C) Immunoblot analysis of SLC7A11, YAP, YAP phosphorylation, and expression of its target genes in WT and MISP-ablated PC-9 cells. (D-E) Immunoblot analysis of YAP phosphorylation, YAP, and CYR61 expression in H1395 (D) and PC-9 (E) cells upon transfection with the indicated siRNA. (F-G) Determination of YAP target genes and SLC7A11 by qPCR in H1395 (F) and PC-9 (G) cells upon transfection with the indicated siRNA. (H) Evaluation of TEAD luciferase reporter activity in 293T cells upon transfection with the indicated siRNA. (I) Determination of YAP ubiquitination in HEK293T cells transfected with Flag-YAP and an empty vector or HA-Ub and indicated siRNA, treated with MG132 (25 μM) for 6 hours, followed by Co-IP with anti-Flag and immunoblotting as indicated. (J) Determination of SLC7A11 and YAP target genes levels in vector and MISP-overexpressing HCC827 cells. (K) Immunoblot analysis of SLC7A11, YAP, YAP phosphorylation, and expression of its target genes in vector and MISP-overexpressing HCC827 cells. (L) Determination of YAP ubiquitination in HEK293T cells co-transfected with Flag-YAP, an empty vector or MISP-V5 along with HA-Ub, treated with MG132 (25 μM) for 6 hours, followed by Co-IP with anti-Flag and immunoblotting as indicated. Data are presented as mean ± SEM. One-way ANOVA was used to compare differences between groups in B, F-H. Unpaired *t*-test was used to determine statistical significance in J. *, **, ***, **** indicate *p* < 0.05, *p* < 0.01, *p* < 0.001, *p* < 0.0001.


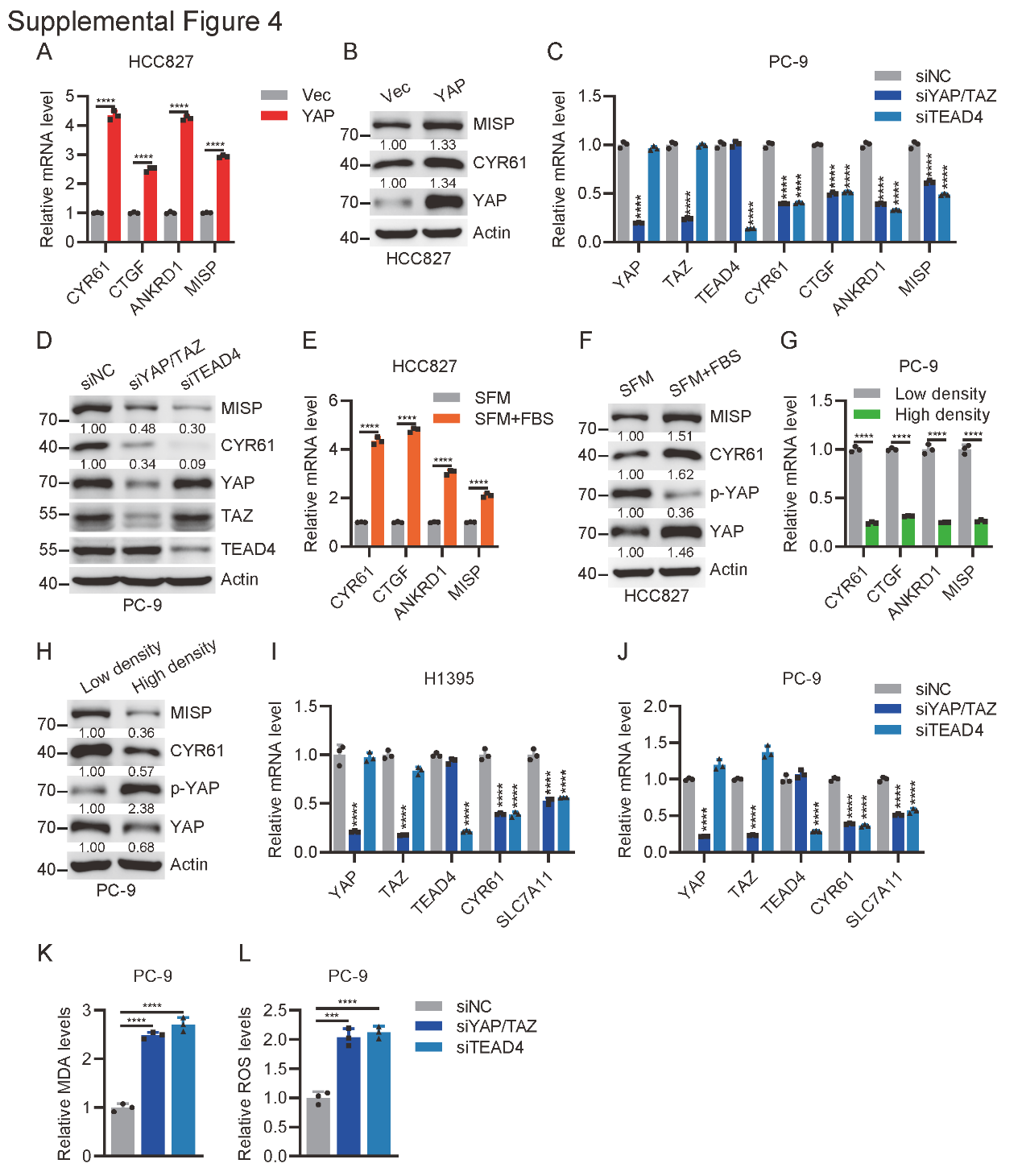


**Supplemental Figure 4: YAP/TEAD4 triggers MISP expression.**

1. Examination of MISP and YAP target genes in HCC827 cells with or without YAP overexpression. (B) Immunoblot analysis of MISP and CYR61 expression in vector and YAP-overexpressing HCC827 cells. (C) Determination of MISP and YAP target genes in PC-9 cells upon YAP/TAZ or TEAD4 depletion. (D) Immunoblot analysis of MISP and CYR61 expression in PC-9 cells upon YAP/TAZ or TEAD4 depletion. (E-F) Determination of the mRNA (E) and protein (F) levels of MISP and YAP target genes in HCC827 cells upon serum starvation or FBS recovery after serum starvation. (G-H) qPCR (G) and immunoblot (H) analysis of MISP and YAP target genes in PC-9 cells at low or high cell density. (I-J) Determination of YAP target genes and SLC7A11 in H1395 (I) and PC-9 (J) cells upon YAP/TAZ or TEAD4 depletion. (K-L) Relative levels of MDA (K) and ROS (L) in PC-9 cells upon YAP/TAZ or TEAD4 depletion. Data are presented as mean ± SEM. Unpaired *t*-test was used to determine statistical significance in A, E, G. One-way ANOVA was used to compare differences between groups in C, I-L. *, **, ***, **** indicate *p* < 0.05, *p* < 0.01, *p* < 0.001, *p* < 0.0001.


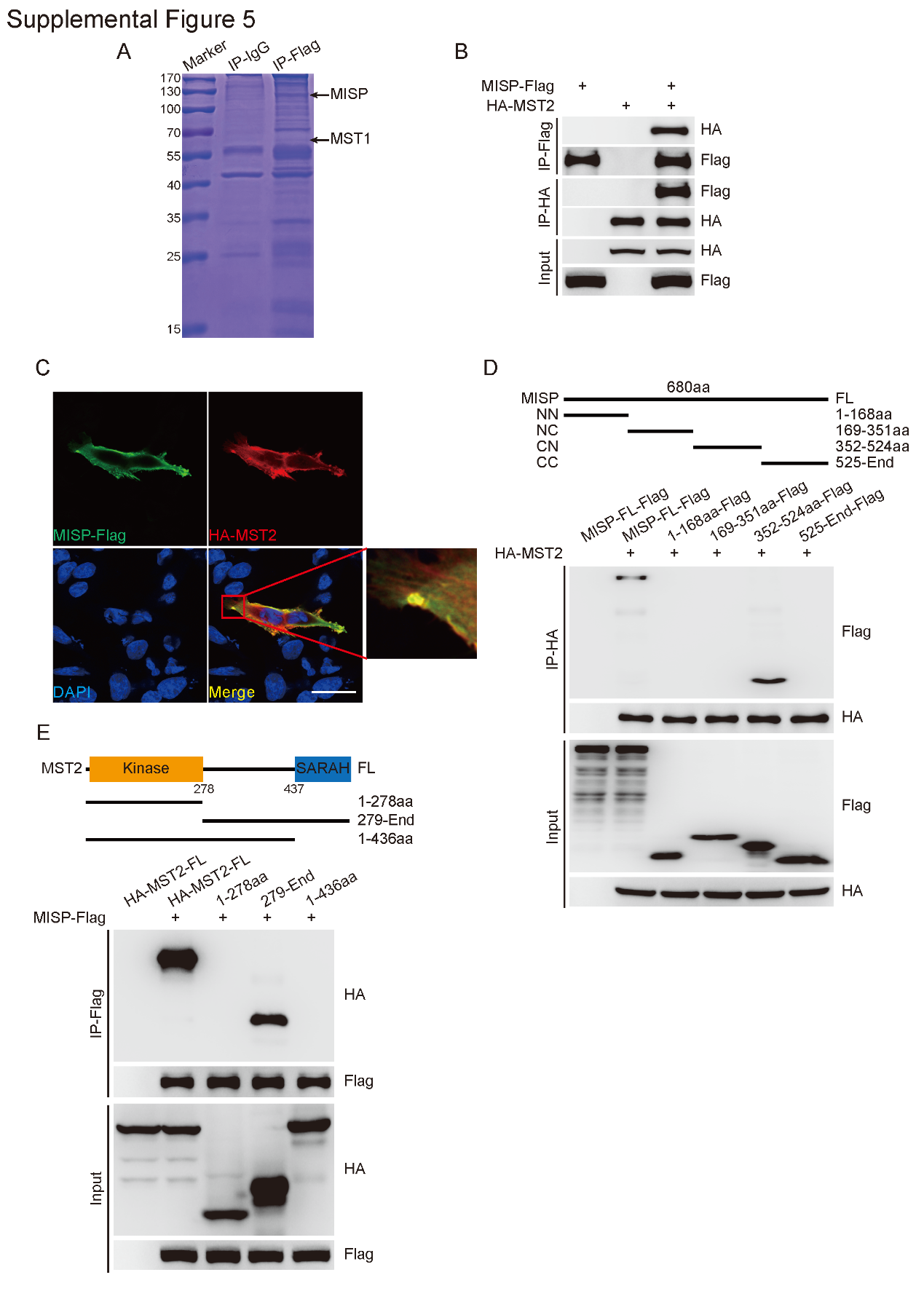


**Supplemental Figure 5: MISP binds to MST2.**

(A) Coomassie brilliant blue staining of MISP interacting partners. PC-9 cells transfected with MISP-Flag were lysed and subjected to immunoprecipitation using anti-IgG or anti-Flag tag antibody. The immunoprecipitates were eluted by boiling in SDS loading buffer and separated by SDS-PAGE before being subjected to Coomassie brilliant blue staining. (B) MISP binds to MST2 in 293T cells. 293T cells were transfected with MISP-Flag and HA-MST2 plasmids alone or in combination for 24 hours, lysed, and subjected to immunoprecipitation using the indicated antibodies. (C) Immunofluorescent staining of MST2 and MISP using the indicated antibodies in H1395 cells. The boxed inset within the left-hand panels points to the specific area depicted in the enlarged image (right panel). Scale bar, 40 μm. (D) MST2 binds to the aa352-524 region of MISP. Diagram of MISP domains (upper). Immunoblotting analysis of MISP fragments using the indicated antibody after Co-IP with anti-HA tag antibody (lower). (E) MISP interacts with the SARAH domain of MST2. Diagram of MST2 motifs (upper). Immunoblot analysis of MST2 fragments using the indicated antibody after Co-IP with anti-Flag tag antibody (lower).


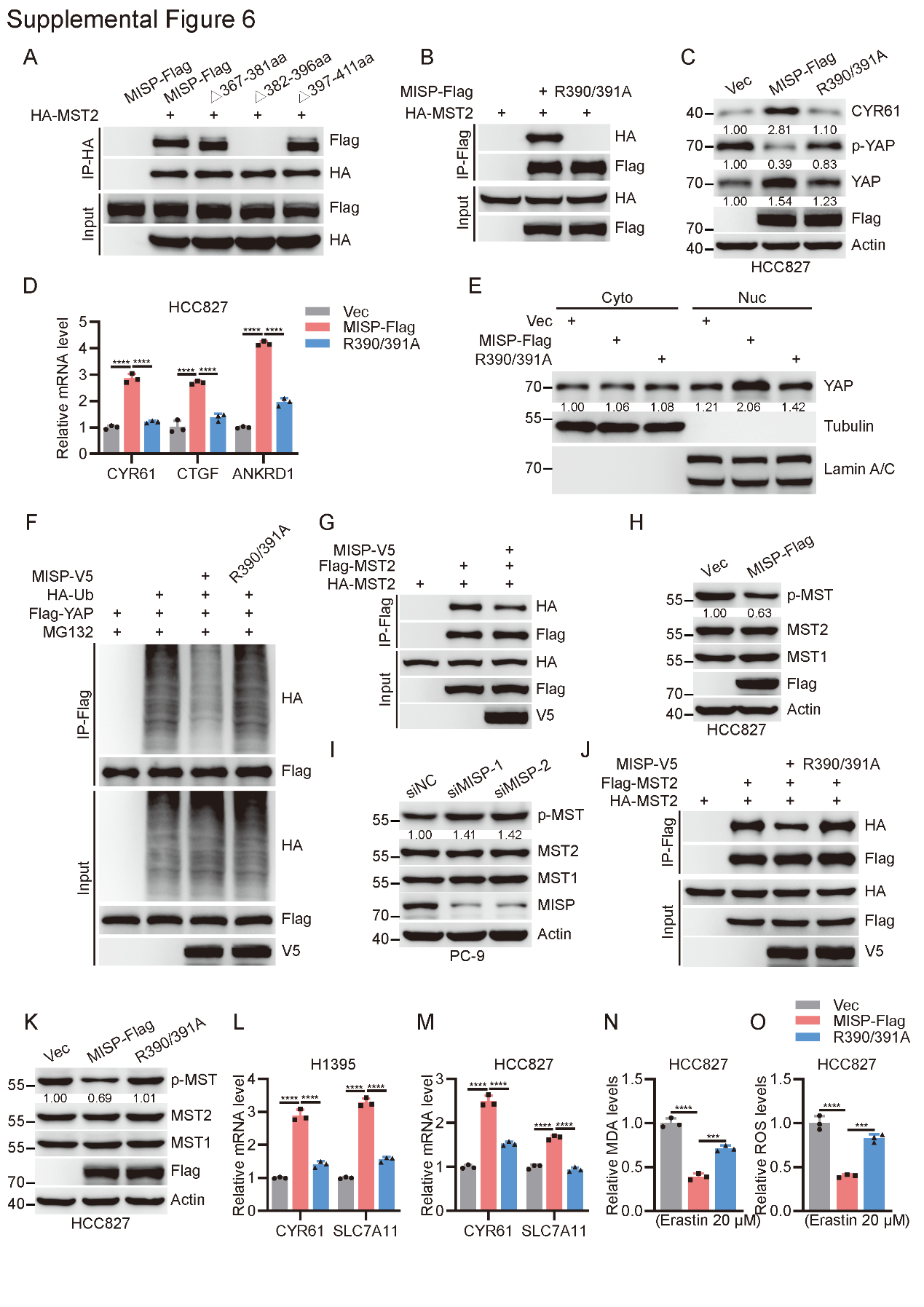


**Supplemental Figure 6: R390/391A mutant abrogates MISP’s function in disrupting MST1/2 dimerization.**

1. Determination of the minimal region required for MISP/MST2 interaction. Indicated deletion mutants were co-transfected with MST2 and subjected to immunoprecipitation. (B) Determination of the binding affinity between WT MISP and the R2A mutant (R390/391A) for MST2. (C) Immunoblot analysis of YAP phosphorylation, YAP, and CYR61 expression in HCC827 cells in the presence of vector, WT MISP, or R2A mutant. (D) Determination of YAP target genes expression in HCC827 cells following expression of vector, WT MISP, or R2A mutant. (E) Cytoplasmic and nuclear fractions derived from vector, WT MISP, or R2A mutant HCC827 cells were subjected to immunoblotting as indicated. (F) Evaluation of YAP ubiquitination in 293T cells upon forced expression of vector, WT MISP, or R2A mutant. (G) Determination of MST2 homodimerization by Co-IP and immunoblot analysis in 293T cells upon forced expression of MISP. (H-I) Immunoblot assessment of MST1 phosphorylation in HCC827 cells upon MISP expression (H) or in PC-9 cells upon MISP ablation (I). (J) Determination of MST2 homodimerization by Co-IP and immunoblot analysis in 293T cells upon forced expression of WT MISP or R2A mutant. (K) Immunoblot analysis of MST1 phosphorylation in HCC827 cells upon forced expression of WT MISP or R2A mutant. (L-M) Determination of CYR61 and SLC7A11 expression in H1395 (L) and HCC827 (M) cells following expression of vector, WT MISP, or R2A mutant. (N-O) Relative levels of MDA (N) and ROS (O) in HCC827 cells following expression of vector, WT MISP, or R2A mutant treated with Erastin (20 μM) for 24 h. Data are presented as mean ± SEM. One-way ANOVA was used to compare differences between groups. *, **, ***, **** indicate *p* < 0.05, *p* < 0.01, *p* < 0.001, *p* < 0.0001.


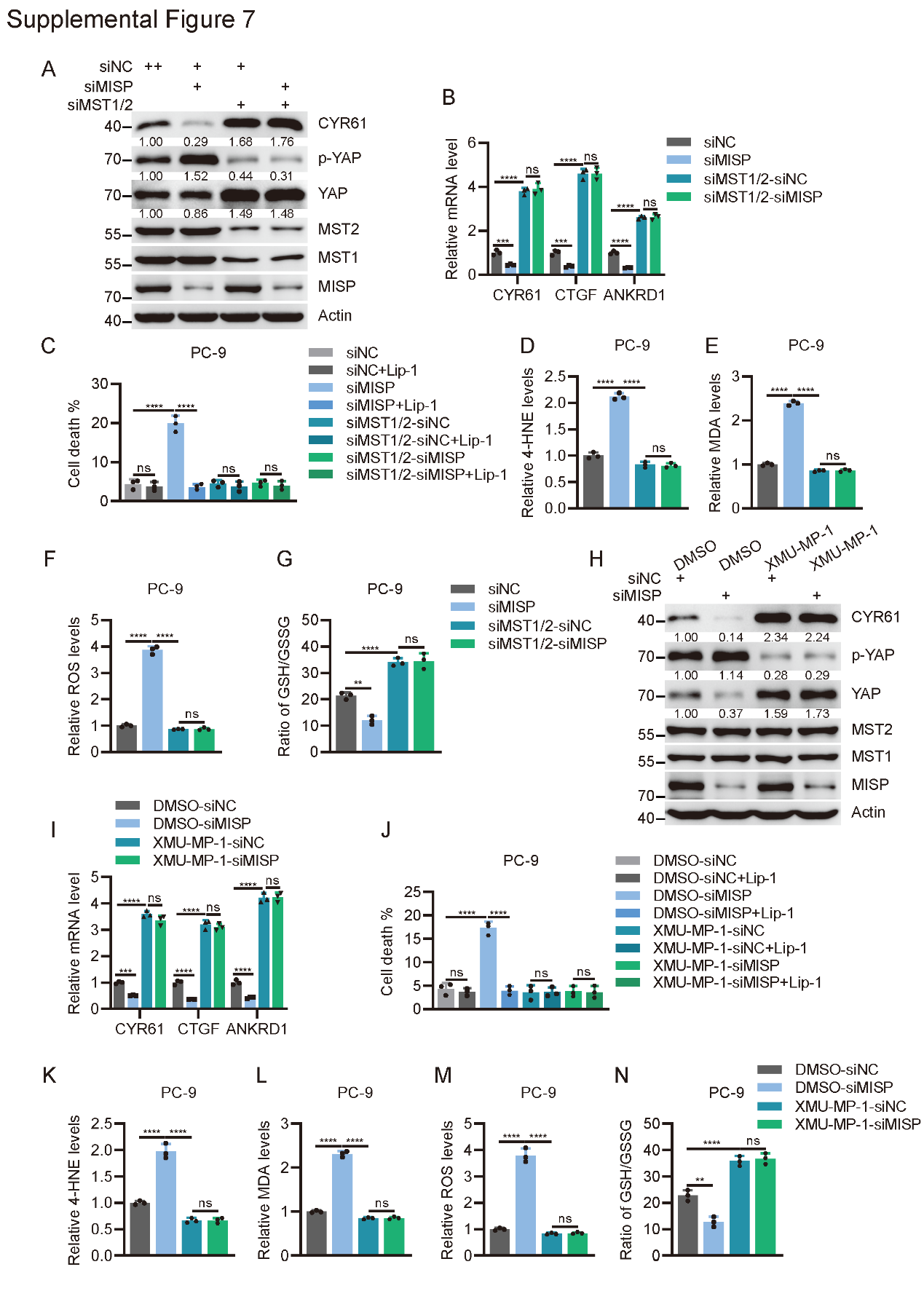


**Supplemental Figure 7: MST1/2 kinases are essential for MISP to trigger YAP activation.**

(A) Immunoblot analysis of YAP phosphorylation, YAP, and CYR61 expression in PC-9 cells upon transfection with the indicated siRNA. (B) Determination of YAP target genes by qPCR in PC-9 cells upon transfection with the indicated siRNA. (C) Relative levels of cell death in PC-9 cells upon transfection with the indicated siRNA treated with DMSO or Lip-1 (500 nM) for 24 h. (D-G) Relative levels of 4-HNE (D), MDA (E), ROS (F) and GSH/GSSG (G) in PC-9 cells upon transfection with the indicated siRNA. (H) Immunoblot analysis of YAP phosphorylation, YAP, and CYR61 expression in PC-9 cells upon transfection with the indicated siRNA with or without XMU-MP-1 treatment (2.5 μM for 12 h). (I) Determination of YAP target genes by qPCR in PC-9 cells following transfection with the indicated siRNA with or without XMU-MP-1 treatment (2.5 μM for 12 h). (J) Relative levels of cell death in PC-9 cells upon transfection with the indicated siRNA with or without XMU-MP-1 (2.5 μM for 12 h) or/and Lip-1 treatment (500 nM for 24 h). (K-N) Relative levels of 4-HNE (K), MDA (L), ROS (M) and GSH/GSSG (N) in PC-9 cells upon transfection with the indicated siRNA with or without XMU-MP-1 treatment (2.5 μM for 12 h). Data are presented as mean ± SEM. One-way ANOVA was used to compare differences between groups. *, **, ***, **** indicate *p* < 0.05, *p* < 0.01, *p* < 0.001, *p* < 0.0001.

Supplementary Table 1. Sequences of siRNA

| Names | Sense (5’ to 3’) | Antisense (5’ to 3’) |
| --- | --- | --- |
| siNC | UUCUCCGAACGUGUCACGUTT | ACGUGACACGUUCGGAGAATT |
| siYAP | GUCAGAGAUACUUCUUAAATT | UUUAAGAAGUAUCUCUGACTT |
| siTAZ | GAUGAAUCAGCCUCUGAAUTT | AUUCAGAGGCUGAUUCAUCGC |
| siTEAD4 | GGAACAAACUGUGCCUGAATT | UUCAGGCACAGUUUGUUCCTT |
| siMST1 | GCCAGAUUGUUGCUAUUAATT | UUAAUAGCAACAAUCUGGCTT |
| siMST2 | GACCAUGAUUGAACAUAAUTT | AUUAUGUUCAAUCAUGGUCTT |
| siMISP-1 | GGACAUAGUACAGGAGACATT | UGUCUCCUGUACUAUGUCCTT |
| siMISP-2 | CCAUCCACCUACACUCAAATT | UUUGAGUGUAGGUGGAUGGTT |

Supplementary Table 2. Sequences of shRNA

| Names | Targeted Sequences (5’ to 3’) |
| --- | --- |
| shLuc | CTTCGAAATGTCCGTTCGGTT |
| shMISP-1 | GACACCAGCTACACATACCAT |
| shMISP-2 | CAGTCATCTGATCTGCTGGAA |

Supplementary Table 3. Sequences of sgRNA

| Names | Targeted Sequences (5’ to 3’) |
| --- | --- |
| sgMISP-1 | ACTGGCCAGCCGTCCCCACG |
| sgMISP-2 | TGGGGACGGCTGGCCAGTGT |

Supplementary Table 4. Primer sequences for qRT-PCR

| Gene names | Forward sequence (5’ to 3’) | Reverse sequence (5’ to 3’) |
| --- | --- | --- |
| *GAPDH* | GAAGGTCGGAGTCAACGG | TCAAAGGTGGAGGAGTGG |
| *MISP* | CCCTGAGCACAAAGCAAGAG | GCAGATCAGATGACTGGGACTT |
| *YAP* | GAACAATGACGACCAATAGCTC | TAGTCCACTGTCTGTACTCTCA |
| *TAZ* | GATCCTGCCGGAGTCTTTCTT | CACGTCGTAGGACTGCTGG |
| *TEAD4* | GAACGGGGACCCTCCAATG | GCGAGCATACTCTGTCTCAAC |
| *CYR61* | AGCCTCGCATCCTATACAACC | TTCTTTCACAAGGCGGCACTC |
| *CTGF* | GAAATGCTGCGAGGAGTG | AGTTGGCTCTAATCATAGTTGG |
| *ANKRD1* | ATCCGACTCCTGATTATGTATGG | GCTATGCGAGAGGTCTTGTAG |
| *SLC7A11* | TCTCCAAAGGAGGTTACCTGC | AGACTCCCCTCAGTAAAGTGAC |

Supplementary Table 5. Primer sequences for CHIP-qPCR

| Names | Forward sequence (5’ to 3’) | Reverse sequence (5’ to 3’) |
| --- | --- | --- |
| MISP-R1 | CAGCTTCACAGACGCGGCTC | CCTTGGCCAGGGAACCCCAT |
| MISP-R2 | GGGCCTCAGTTTTCCCCGATG | TGTGAAAGCTCAGGGTGCGCG |
| MISP-R3 | TCCCCACAGTCCCAGCAAGG | CAGTGAGCCGAGATCACACCAC |
| CTGF | GGCCAAGTTGCCTTTAATACACATG | CCTCTGAAGCTCAGGAAGAATTTTCG |
